# DNA methylation landscape reveals *LIN7A* as a decitabine-responsive marker in patients with t(8;21) acute myeloid leukemia

**DOI:** 10.1186/s13148-023-01458-0

**Published:** 2023-03-03

**Authors:** Shujiao He, Yan Li, Xuanren Shi, Lei Wang, Diya Cai, Jingfeng Zhou, Li Yu

**Affiliations:** 1 Department of Hematology and Oncology, Shenzhen University General Hospital, International Cancer Center, Shenzhen Key Laboratory, Hematology Institution of Shenzhen University, Shenzhen University Medical School, Shenzhen University, Shenzhen, 518000 China; 2 Department of Hematology, Peking Third Hospital, Beijing, 100191 China; 3 Department of Hematology, Chinese People’s Liberation Army General Hospital, Beijing, 100853 China

**Keywords:** Acute myeloid leukemia, Decitabine, Differentially methylated region, DNA methylation sequencing, LIN7A, t(8;21) translocation

## Abstract

**Background:**

Despite its inconsistent response rate, decitabine, a demethylating agent, is often used as a non-intensive alternative therapeutic agent for acute myeloid leukemia (AML). It has been reported that relapsed/refractory AML patients with t(8;21) translocation achieved better clinical outcomes with a decitabine-based combination regimen than other AML subtypes; however, the mechanisms underlying this phenomenon remain unknown. Herein, the DNA methylation landscape of de novo patients with the t(8;21) translocation was compared with that of patients without the translocation. Moreover, the methylation changes induced by decitabine-based combination regimens in de novo*/*complete remission paired samples were investigated to elucidate the mechanisms underlying the better responses observed in t(8;21) AML patients treated with decitabine.

**Methods:**

Thirty-three bone marrow samples from 28 non-M3 AML patients were subjected to DNA methylation sequencing to identify the differentially methylated regions and genes of interest. TCGA-AML Genome Atlas-AML transcriptome dataset was used to identify decitabine-sensitive genes that were downregulated following exposure to a decitabine-based regimen. In addition, the effect of decitabine-sensitive gene on cell apoptosis was examined in vitro using Kasumi-1 and SKNO-1 cells.

**Results:**

A total of 1377 differentially methylated regions that specifically responsive to decitabine in t(8;21) AML were identified, of which 210 showed hypomethylation patterns following decitabine treatment aligned with the promoter regions of 72 genes. And the methylation-silencing genes, *LIN7A*, *CEBPA*, *BASP1*, and *EMB* were identified as critical decitabine-sensitive genes in t(8;21) AML. Moreover, AML patients with hypermethylated *LIN7A* and reduced *LIN7A* expression had poor clinical outcomes. Meanwhile, the downregulation of LIN7A inhibited decitabine/cytarabine combination treatment-induced apoptosis in t(8;21) AML cells in vitro.

**Conclusion:**

The findings of this study suggest that *LIN7A* is a decitabine-sensitive gene in t(8;21) AML patients that may serve as a prognostic biomarker for decitabine-based therapy.

**Supplementary Information:**

The online version contains supplementary material available at 10.1186/s13148-023-01458-0.

## Background

Administration of intensive chemotherapy with cytarabine (Ara-C) and anthracycline has profoundly improved the clinical outcomes of patients with acute myeloid leukemia (AML) [1]. However, given that the application of this combination treatment is heavily affected by several clinical characteristics of patients, including age, performance status, and underlying medical comorbidities, many patients are ineligible for such an aggressive regimen [2]. Hypomethylating agents, such as decitabine (DAC) and azacytidine, are effective low-intensity alternatives for treating patients with a low-performance status and comorbidities [3]. However, there are discrepancies in the clinical outcomes among various DAC-based clinical trials. In fact, it has been reported that the response rate of DAC monotherapy in de novo AML patients varies from 14 to 47% [4, 5]. Although combination therapy with DAC and low-dose Ara-C, or molecularly targeted therapies has improved the clinical outcomes of these patients, several patients remain resistant to DAC-based therapy [6, 7]. Hence, the ability to identify DAC-sensitive patients would aid clinical decision-making and help improve patient response rates. Notably, a single-arm clinical trial revealed that t(8;21) AML is more sensitive to DAC-based chemotherapy than other AML subtypes [8].t(8;21)(q22;q22) is the most common chromosomal translocation in patients with AML, accounting for 10–20% of the total AML cases [9, 10]. Derived from the t(8;21) translocation, the AML1-ETO (AE) fusion protein is primarily associated with enhanced self-renewal and impaired hematopoietic stem cell differentiation [10]. However, this fusion protein is also reported to be involved in DNA methylation regulation through the recruitment of DNA methylation transferase [11]. Indeed, based on DNA methylation sequencing, t(8;21) AML constitutes a distinctive subgroup of AML [12, 13]. Thus, we hypothesized that a specific DNA methylation signature may condition DAC response in patients with t(8;21) AML. To address this, we explored DNA methylation sequencing in patients with non-M3 AML to identify differentially methylated regions (DMRs) and corresponding genes sensitive to DAC.

## Methods

### Study cohort

Twenty-eight non-M3 AML patients who were monitored at the Hematology Department of the Chinese People’s Liberation Army General Hospital between August 2014 and June 2016 were enrolled in this study and underwent DNA methylation sequencing, as described previously [13]. Three of these patients had the t(8;21) translocation. A methylation sequencing dataset comprising 33 bone marrow samples was generated, including 23 non-paired de novo samples, 2 paired t(8;21) de novo/complete remission (CR) samples, and 3 paired non-t(8;21) de novo/CR samples. All de novo AML samples were collected before treatment, while CR samples were obtained after the first round of treatment with the DCAG regimen, which involved decitabine (20 mg/m^2^ on days 1–5), Ara-C (10 mg/m^2^ every 12 h on days 1–5), aclarubicin (20 mg on days 1, 3, and 5), and granulocyte colony-stimulating factor (300 μg/d from d0 to neutrophil recovery). All patients were diagnosed and assessed according to the AML guidelines of the National Comprehensive Cancer Network (version 1.2017; http://www.nccn.org/). As described previously [13], specimen collection was conducted only after written informed consent was obtained from each participant. Patient characteristics are summarized in Tables 1 and 2.

**Table 1:** Characteristics of the de novo patient study cohort (*n* = 28)

Characteristic	Value
Age at diagnosis, years	49.07 ± 17.94
Sex, no. (%)	
Male	11 (39)
Female	17 (61)
Bone marrow blast, no. (%)	67.55 ± 23.74
AML FAB subtype, no. (%)	
M1	1 (3.57)
M2	5 (17.86)
M4	10 (35.71)
M5	11 (39.29)
M6	1 (3.57)
2017 NCCN cytogenetic risk classification, no. (%)	
Good	5 (17.86)
Intermediate	20 (71.43)
Poor	3 (10.71)
2017 NCCN molecular risk classification, no. (%)	
Good	12 (42.86)
Intermediate	7 (25.00)
Poor	9 (32.14)
Mutation, no. (%)	
t(8;21)	3 (10.71)
inv(16)	2 (7.14)
* NPM1*	4 (14.29)
* FLT3*	6 (21.43)
* DNMT3A*	3 (10.71)
* IDH1* or *IDH2*	4 (14.29)
* KRAS* or *NRAS*	4 (14.29)
* TP53*	2 (7.14)
bi*CEBPA*	7 (25.00)

**Table 2:** Characteristics of the de novo/CR paired samples (*n* = 5)

	Age (years)	Sex	Bone marrow blast at diagnosis %	Cytogenetics at diagnosis	Risk classification	Induction regimen
Pair 1	59	Female	45.2	Normal karyotype	Intermediate	DCAG
Pair 2	34	Female	67.2	46, XX, inv(16)	Good	DCAG
Pair 3	60	Female	56.4	46, XX, t(11;20)	Intermediate	DCAG
Pair 4	73	Female	81	46, XX, t(8;21)	Good	DCAG
Pair 5	50	Female	83.2	45, XX, − X, t(8;21)	Good	DCAG

### DNA methylation sequencing

For the enrichment of mononuclear cells, bone marrow samples were separated using a Ficoll density gradient (Sigma-Aldrich, St. Louis, MO, USA), and DNA from the lymphoblasts was purified using a Wizard Genomic DNA Purification Kit (Promega, Madison, WI, USA). MethylC-capture sequencing was performed as described previously [13]. Briefly, an amplified bisulfate-converted DNA fragment library was constructed using a SeqCap Epi enrichment system (Roche NimbleGen, Madison, WI, USA), and a HiSeq2500 system (Illumina, San Diego, CA, USA) was used for DNA sequencing.

### Methylation profiling

Following the removal of the adapter sequences and poor-quality reads based on FastQC (https://www.bioinformatics.babraham.ac.uk/index.html), Bismark [14] (v0.10.1; parameters: -pe, -bowtie2, -directional, -unmapped) was used to map methylation regions to the GRCh37 human assembly. The methylation level at each site was calculated by dividing the methylated reads by the total reads. DMRs were confirmed using Metilene [14] (v0.2-6; parameters: -M 300, -m 5, -d 0.2, -f 1, -t 1). DMRs exhibiting changes ≥ 20% were subjected to sequential analysis. Recurrent or unique DMRs between different groups were confirmed using Bedtools [15] (v2.25.0, https://bedtools.readthedocs.io/en/latest/; parameters: intersect -a DMRa.bed, -b DMRb.bed, -wa). The differences in absolute methylation levels in the DMRs were determined by subtracting the methylation level of the non-t(8;21) samples from that of t(8;21) or CR samples from de novo samples. DMRs with absolute methylation differences > 0 were defined as being hypermethylated, whereas those with absolute methylation differences < 0 were defined as being hypomethylated. “Promoter” regions were identified as DNA regions between 2200 bp upstream and 500 bp downstream of the transcription start sites (TSS). DNA regions only overlapping the gene body were identified as “gene body,” while those partially overlapping the promoter and partially overlapping gene body were identified as “Promoter/Gene body (P/Gb).” The remaining DNA regions were identified as “Other.”

### Acquisition of in silico datasets

Clinical and RNA sequencing data from 156 patients with AML (TCGA-AML), including 7 t(8;21) AML and 149 non-t(8;21) AML patients, were obtained from the UCSC Xena dataset platform (https://xenabrowser.net/datapages/). R (V3.6.1) and the *limma* package were used to identify differentially expressed genes related to t(8;21) AML.

GSE18700, a methylation profile containing data from 24 t(8;21) and 320 non-t(8;21) AML patients and constructed using a methylation array targeting the promoter regions, was obtained from the Gene Expression Omnibus database (https://www.ncbi.nlm.nih.gov/gds). The methylation data, defined based on GRCh36Refseq, were remapped to GRCh37Refseq using ANNOVAR [16] to make the data objectively comparable to those of our real-world study.

### Cell culture and treatment

Kasumi-1, SKNO-1, U937, and K562 cell lines were purchased from the American Type Culture Collection (Manassas, VA, USA) and maintained in Gibco RPMI-1640 (Thermo Fisher Scientific, Waltham, MA, USA) supplemented with streptomycin (100 µg/mL, Gibco), penicillin (50 U/mL, Gibco), and 10% fetal bovine serum (FBS; Gibco). As previously described [13], SKNO-1-siAE cell line was generated by transfection with a lentiviral vector encoding siAGF1 oligonucleotides against the AML1-ETO mRNA fusion site to silence the expression of the AML1-ETO protein in SKNO-1 cells [17]. Cells were cultured in plastic tissue culture plates in a humidified 5% CO_2_ atmosphere at 37 °C. SKNO-1-siAE cell were cultured for a month before sequencing. Lyophilized DAC (Topscience, Shanghai, China) and Ara-C (Topscience) were dissolved in dimethyl sulfoxide (Thermo Fisher Scientific) and stored at − 80 °C.

### Lentivirus packaging and infection

Human embryonic kidney 293 T cells (ATCC) were maintained in Dulbecco’s modified Eagle’s medium containing streptomycin (100 µg/mL), penicillin (50 U/mL), and 10% FBS. Short hairpin RNAs (shRNAs; Sigma-Aldrich) were cotransfected into 293 T cells with pHR and VSVG plasmids to produce shRNA lentiviruses. Kasumi-1 and SKNO-1 cells were transfected with control shRNA lentiviruses (shNC) or lentiviruses targeting *LIN7A* (shLIN7A).

### Cell viability and apoptosis assays

Cell viability was assessed using the MTS assay (G1111; Promega) following treatment with different concentrations of DAC for 7 days; the optical density was measured at 490 nm. Cell viability was calculated using the following formula: cell viability (%) = (Abs_treated blank_/Abs_control blank_) × 100%.

After successfully transfecting cells with shNC or shLIN7A lentivirus, the cells were treated with 10 nM DAC for 3 days; the medium was then replaced with fresh medium. On day 4, 2 × 10^5^ cells/well were seeded into 6-well plates and either left untreated or treated with 100 nM Ara-C for an additional 3 days. Thereafter, on day 7, the cells were harvested and stained with annexin V-FITC (BD Biosciences, Franklin Lakes, NJ, USA) for 15 min at room temperature in the dark. Propidium iodide was added to the samples before analysis to distinguish live cells from dead ones, and the apoptosis rate was determined using a CytExpert flow cytometer (Beckman Coulter Life Sciences, Indianapolis, IN, USA). Apoptosis experiments were performed in triplicate using the following treatment groups: DAC alone, Ara-C alone, and DAC followed by Ara-C.

### DNA extraction and methylation-specific polymerase chain reaction (PCR)

Genomic DNA from Kasumi-1 and SKNO-1 cells was isolated using a Wizard Genomic DNA Purification Kit (Promega). Furthermore, genomic DNA was treated with sodium bisulfate (EpiTect Bisulfite Kit; Qiagen, Hilden, Germany) according to the manufacturer’s instructions. Methylation-specific PCR primers were designed using MethPrimer (http://www.urogene.org/methprimer/) and are listed in Additional file 1: Table S1. GoTaq Green Master Mix (Promega) was used for methylation-specific PCR according to the manufacturer’s instructions. PCR products were analyzed via electrophoresis on a 2% agarose gel.

### RNA extraction and quantitative PCR

Invitrogen TRIzol reagent (Thermo Fisher Scientific) was used to extract total RNA. cDNA was synthesized using a cDNA kit (Takara Bio, Kusatsu, Japan) according to the manufacturer’s instructions. An ABI PRISM 7500 Sequence Detection System (Thermo Fisher Scientific) was used to perform quantitative reverse transcription-PCR (qRT-PCR) under the following cycle conditions: 95 °C for 30 s to denature the cDNA template, followed by 40 cycles of 95 °C for 5 s and 60 °C for 20 s. Gene expression levels were calculated using the 2^−ΔΔCt^ method. The primers used for qRT-PCR are listed in Additional file 1: Table S2. Independent experiments were performed in triplicate.

### Western blotting

Radioimmunoprecipitation assay buffer, supplemented with phenylmethylsulfonyl fluoride and phosphatase inhibitors (Roche, Basel, Switzerland), was used for cell lysis. Protein concentration was determined using a BCA protein assay kit (Thermo Fisher Scientific). Subsequently, the proteins were subjected to sodium dodecyl sulfate–polyacrylamide gel electrophoresis and transferred onto polyvinylidene fluoride membranes. The membranes were incubated overnight at 4 °C with the following primary antibodies: anti-LIN7A (1:1000; ab174297, Abcam, Cambridge, UK), anti-DNMT3A (1:1000; ab188470, Abcam), and anti-GAPDH (1:10,000; RM2002, Beijing Ray Antibody Biotech, Beijing, China). Thereafter, the membranes were washed with Tris-buffered saline with Tween 20 and then incubated with the relevant secondary antibodies (31,463 and 31,437, Thermo Fisher Scientific) for 2 h at room temperature.

### Statistical analysis

All data were expressed as the mean ± standard error of the mean from at least three independent experiments. SPSS software (version 20.0; SPSS Inc., Chicago, IL, USA) was used to perform all statistical analyses. Student’s *t*-test and one-way analysis of variance were used to determine the statistical differences among the experimental groups. All statistical tests were two-sided, statistical significance was defined as *p* < 0.05.

## Results

### Identification of DMRs specific to t(8;21) AML

**Fig. 1:**
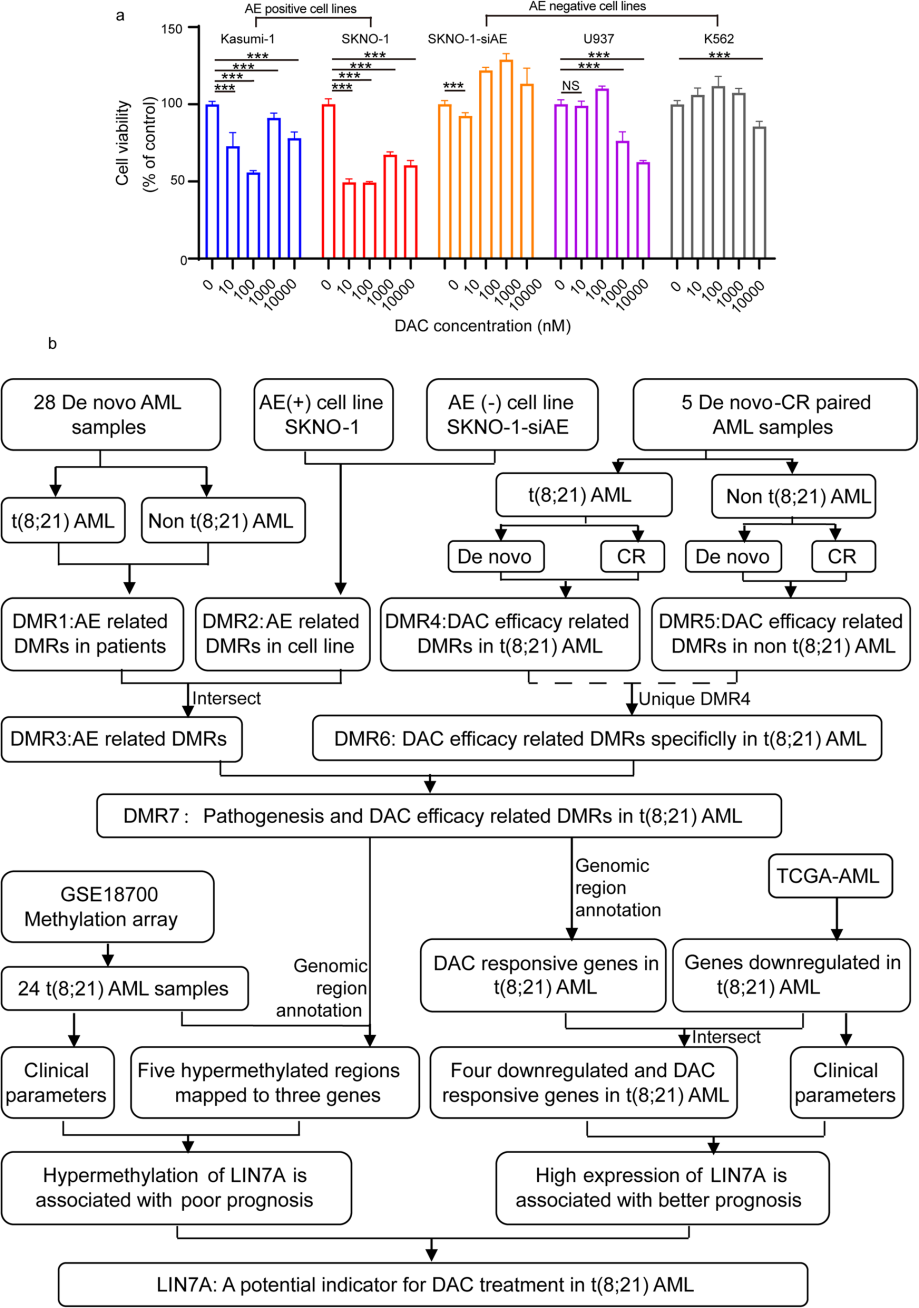
**a** Comparison of the inhibition effect of different decitabine (DAC) concentrations on acute myeloid leukemia (AML) cell lines with (AE positive) or without (AE negative) t(8;21) translocation. **b** Study flow chart. Overview of the study cohort and follow-up analyses to identify DAC-sensitive regions in t(8;21) AML

Considering that patients with relapse/refractory t(8;21) AML have been reported to more likely benefit from a DAC-based regimen [8], we sought to further determine the effect of DAC on t(8;21) AML cells by initially comparing the viability of t(8;21) and non-t(8;21) AML cells upon treatment with DAC. The results showed that DAC significantly inhibited cell viability in t(8;21) AML cell lines (Kasumi-1 and SKNO-1) at low dosages of 10 and 100 nM (Fig. 1a).

**Fig. 2:**
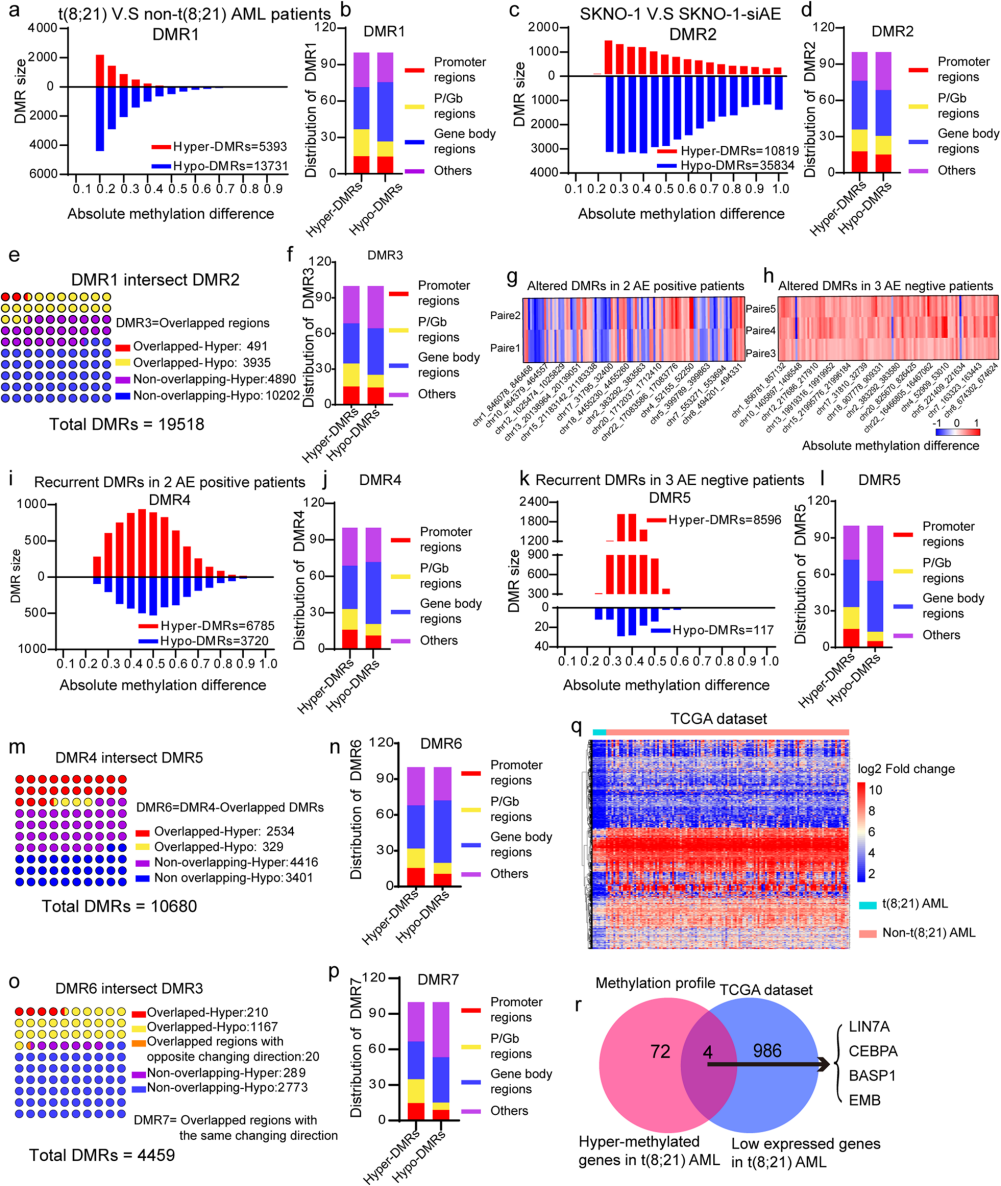
Characterization of DMR1–DMR7. **a** DMRs between 3 t(8;21) and 25 non- t(8;21) acute myeloid leukemia (AML) samples. A total of 5393 hypermethylated regions [absolute methylation level of t(8;21)—non-(t8;21) AML > 0.2; hypermethylated DMR1] and 39 hypomethylated regions [absolute methylation level of t(8;21)—non-(t8;21) AML <  − 0.2; hypomethylated DMR1] were identified in the t(8;21) AML samples. **b** Genomic distribution of DMR1. **c** DMRs (DMR2) between the AE-positive SKNO-1 and AE-negative SKNO-1-siAE cell lines. A total of 10,819 hypermethylated DMR2 [absolute methylation level of SKNO-1—SKNO-1-siAE > 0.2] and 35,834 hypomethylated DMR2 [absolute methylation level of SKNO-1—SKNO-1-siAE < − 0.2] were identified. **d** Genomic distribution of DMR2. **e** Alignment of DMR2 with DMR1. DMR regions that both identified as DMR1 and DMR2 were recognized as DMR3. **f** Genomic distribution of DMR3. **g**–**h** Methylation profile alteration after DAC-based regimen treatment of **g** t(8;21) and **h** non-t(8;21) AML patients. **i** DMRs with the same change direction after treatment in the 2 t(8;21) AML patients were identified as DMR4, comprising 6875 hypermethylated DMR4 [absolute methylation level of de novo sample–CR sample > 0.2] and 3720 hypomethylated DMR4 [absolute methylation level of de novo sample–CR sample <  − 0.2]. **j** Genomic distribution of DMR4. **k** DMRs with the same change direction after treatment in the 3 non-t(8;21) AML patients were identified as DMR5, comprising 8596 hypermethylated DMR5 [absolute methylation level of de novo sample–CR sample > 0.2] and 117 hypomethylated DMR [absolute methylation level of de novo sample–CR sample <  − 0.2]. **l** Genomic distribution of DMR5. **m** Alignment of DMR5 with DMR4. DMR regions that only appeared in t(8;21) AML patients (DMR4) comprised DMR6. **n** Genomic distribution of DMR6. **o** Alignment of DMR3 with DMR6. Overlapped regions with the same change direction were identified as DMR7. **p** Genomic distribution of DMR7. **q** A cluster of downregulated genes in patients with the t(8;21) translocation compared with those without, according to TCGA-AML dataset. **r** Four genes were obtained through the intersection of the 72 hypermethylated genes derived from DMR7 with the 986 downregulated genes in t(8;21) AML. *CR* Complete response; *DAC* Decitabine

The scheme of this study is illustrated in Fig. 1b. Initially, to determine the distinctive methylation patterns in t(8;21) AML, the methylation profiles of 3 de novo t(8;21) AML samples were compared to those of 25 non-t(8;21) AML samples. A total of 19,124 DMRs were identified and collectively designated as the “DMR1” profile. The frequency of t(8;21)-related hypermethylated DMRs within DMR1 (*n* = 5393; 28.21%) was much lower than that of hypomethylated DMRs (*n* = 13,731; 71.79%; Fig. 2a). Moreover, the genomic distribution of hypermethylated and hypomethylated DMRs was preferentially enriched in gene body regions (34.75% and 48.86%, respectively). In contrast, 14.49% and 14.16% of the hypermethylated and hypomethylated DMRs were in promoter regions, respectively (Fig. 2b).

We also compared the methylation signatures of the t(8;21) cell line SKNO-1 with an AML1-ETO knockdown cell line (SKNO-1-siAE), and the DMRs were collectively designated as the “DMR2” profile. DMR2 contained more DMRs (*n* = 46,653) than DMR1. However, similar to DMR1, hypomethylated DMRs within DMR2 (*n* = 35,834) were more frequent than hypermethylated DMRs (*n* = 10,819; Fig. 2c). Likewise, 40.34% of hypermethylated DMRs and 37.95% of hypomethylated DMRs were found in the gene body regions, and 17.84% and 15.23% were mapped to promoter regions (Fig. 2d). These results indicated that downregulation of AML1-ETO may reverse the methylation patterns of AML, particularly in non-promoter regions.

To refine the methylation signature associated with t(8;21) translocation, we aligned the DMR2 profile with that of DMR1 (Fig. 2e). As expected, no overlapping DMRs with opposite change directions were identified (Fig. 2e). Recurrent DMRs with the same change direction between the two datasets were collectively named “DMR3” profile. DMR3 comprised 491 hyper- and 3935 hypo-methylated regions (Fig. 2e). Gene body regions remained the most affected across the genome, containing 33.81% and 39.14% of the hyper- and hypo-methylated DMRs within DMR3, respectively. The promoter regions contained 15.27% and 14.41% of the hyper- and hypo-methylated DMRs within DMR3, respectively (Fig. 2f). In brief, DMR3 was considered the t(8;21)-associated methylation signature.

### Identification of DAC-responsive DMRs in t(8;21) AML

To examine the effects of the DAC-based regimen on the methylation landscape of AML patients with or without the t(8;21) translocation, we sequentially compared the differential methylation patterns of the two t(8;21) de novo/CR-paired samples with those of the three non-t(8;21) de novo/CR-paired samples. These five patients achieved CR after administration of one cycle of the DCA-based regimen (Table 2, Fig. 2g, h).

DMRs between the two paired t(8;21) de novo/CR samples were preferentially interrogated (Fig. 2g). As expected, no recurrent DMRs with opposite change directions were observed. Recurrent DMRs with the same change direction across the two paired samples were collectively designated “DMR4” (Fig. 2g and i). Of the hypermethylated DMRs within DMR4, 64.59% had reduced methylation levels after treatment with the DAC-based regimen (Fig. 2i). Hyper- (35.58%) and hypo-methylated (51.02%) DMRs within DMR4 were preferentially enriched in gene body regions. In contrast, 15.95% and 11.26% of hyper- and hypo-methylated DMRs, respectively, were mapped to promoter regions (Fig. 2j).

Recurrent DMRs with the same change direction across the three non-t(8;21) paired samples were collectively designated “DMR5” (Fig. 2h, k, l). DMR5 contained 8596 hypermethylated and 117 hypomethylated DMRs. Strikingly, in non-t(8;21) paired samples, the proportion of hypermethylated DMRs with reduced methylation levels after treatment accounted for up to 98.66% of the DMR5 profile, while hypermethylated DMRs with increased methylation levels accounted for only 1.34% (Fig. 2k). In addition, DMR5 patterns were primarily localized in gene body regions, with hyper- and hypo-methylated DMRs accounting for 38.82% and 41.88% of the DMR5 profile, respectively (Fig. 2l).

By using DMR4 as the reference DNA set, we aligned DMR5 with DMR4 profiles to identify shared and unique DMRs (Fig. 2m). Recurrent DMRs with the same change direction between DMR4 and DMR5 were considered non-specific methylation changes induced by the DAC-based regimen; however, DMRs unique to DMR4 (Fig. 2m, n) were considered DAC-responsive DMRs in t(8;21) AML, and were collectively designated as “DMR6” (Fig. 2m). Overall, 7817 unique DMRs were identified, including 4416 (56.49%) hyper- and 3401 (43.62%) hypo-methylated DMRs (Fig. 2m). As shown in the DMR6 localization map (Fig. 2n), promoter regions contained 686 (15.34%) hyper- and 419 (10.70%) hypo-methylated DMRs, while gene bodies contained 1593 hyper- (36.07%) and 1777 hypo-methylated (52.25%) DMRs.

### Identification of DMRs specifically related to the DAC response in t(8;21) AML

To effectively identify DAC-responsive DMRs in patients with t(8;21) AML, we compared the specific methylation profiles of t(8;21) AML (DMR3; Fig. 2e) with those of the DAC-responsive DMRs (DMR6; Fig. 2m). Consequently, 1377 overlapping DMRs with the same change direction were identified and collectively designated “DMR7” (Fig. 2o). Twenty overlapping DMRs with opposite change directions were eliminated. DMR7 contained 210 (15.25%) hypermethylated regions that exhibited reduced methylation levels after treatment, while comprising 1167 (84.75%) hypomethylated regions with increased methylation levels after treatment (Fig. 2o, p). Given that hypermethylated regions are more readily detected in clinical practice, the 210 hypermethylated DMRs within DMR7 were selected for further analysis. Among these, 31 (14.76%) were localized to promoter regions and 42 (20.00%) were localized to P/Gb sequences (Fig. 2p) that were aligned with the promoter of 72 genes (see Additional file 1: Table S3). Hence, these genes that were hypermethylated in promoter regions and may be involved in the DAC response in t(8;21) AML were subjected to subsequent analysis.

### Identification of genes specifically related to the DAC response in t(8;21) AML

**Fig. 3:**
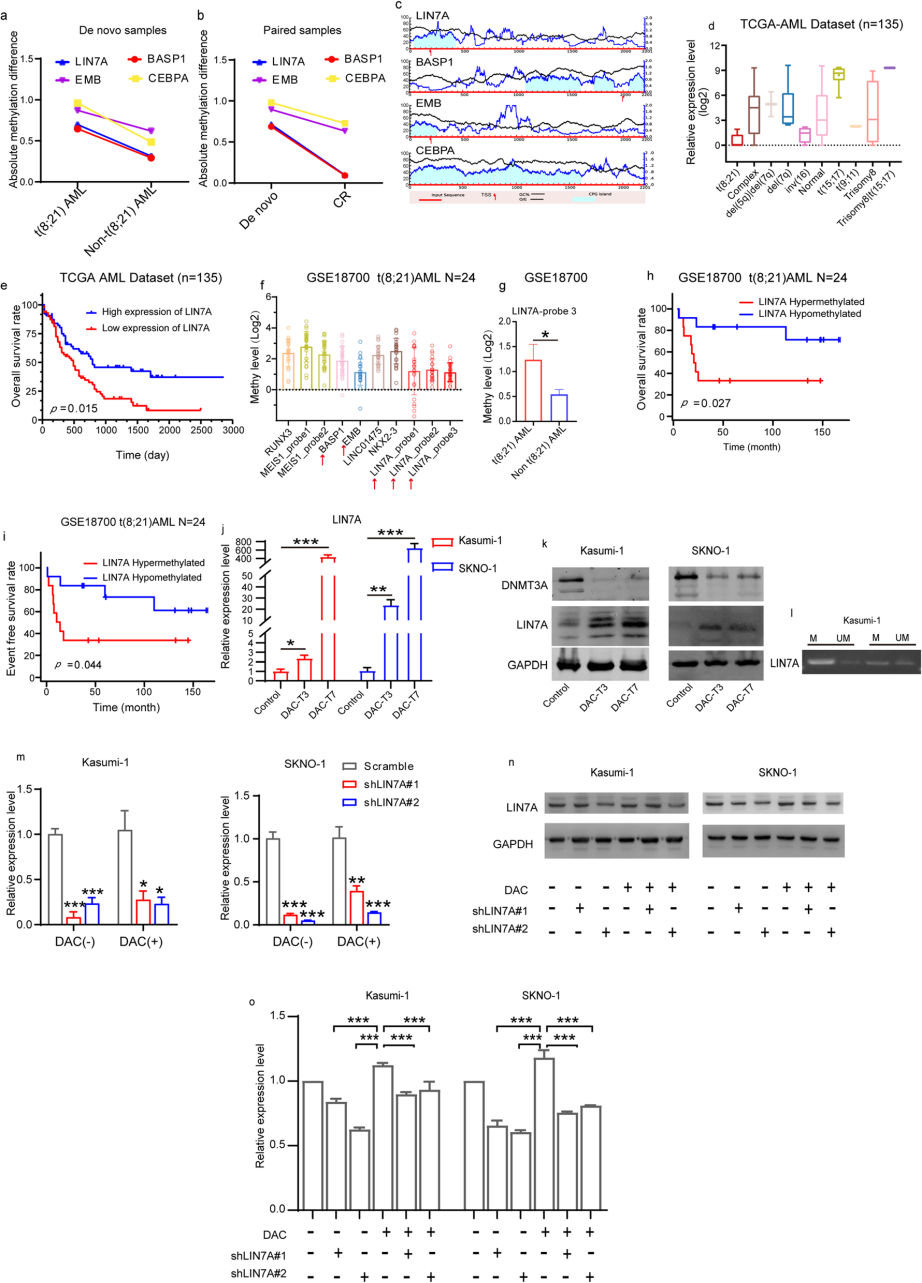
**a** Absolute methylation levels of LIN7A, BASP1, EMB, and CEBPA in t(8;21) AML patients were higher than those in non-t(8;21) AML patients. **b** Absolute methylation levels of LIN7A, BASP1, EMB and CEBPA in de novo samples were higher than those in CR samples. **c** Distribution of CpG islands in the promoter region of the four genes. **d** Expression of LIN7A was much lower in patients with the t(8;21) translocation compared with that in other karyotypes according to TCGA dataset. **e** The expression levels of LIN7A positively correlated with the overall survival rate of AML patients. **f** LIN7A, BASP1, and EMB were identified as methylated genes in t(8;21) AML patients according to the GSE18700 dataset. **g** The methylation levels of LIN7A-probe 3 targeted sequence were significantly higher in patients with t(8;21) AML. **h**–**i** The methylation levels of the LIN7A-probe 3 targeted sequence negatively correlated with the **h** overall and **i** event-free survival rates of patients with t(8;21) AML. **j** Quantitative real-time polymerase chain reaction and **k** western blotting analyses showed increased mRNA and protein levels of LIN7A after treatment with 10 nM DAC for three (DAC-T3) or seven (DAC-T7) consecutive days. **l** Methylation-specific PCR of Kasumi-1 cells showed methylation of the probe 3-targeted sequence by two different paired primers. **m** LIN7A expression analysis determined by qRT-PCR after treatment with shLIN7A lentiviruses accompanied with or without 10 nM DAC in Kasumi-1 and SKNO-1 cells. **n** Western blot analysis of LIN7A expression change after treatment with shLIN7A lentiviruses accompanied with or without 10 nM DAC in Kasumi-1 and SKNO-1 cells. *DMR* Differentially methylated region; *DAC* Decitabine. *p < 0.05, **p < 0.01, and ***p < 0.001

It is well known that methylation of promoter sequences results in repressed gene expression. Therefore, TCGA-AML transcriptome dataset was used to further refine DAC-responsive genes in t(8;21)AML. A total of 128 non-M3 AML samples, including 7 t(8;21) AML samples, were analyzed. Upon comparing the expression profiles of t(8;21) and non-t(8;21) AML samples, 986 downregulated genes in t(8;21) AML were identified (Fig. 2q; see Additional file 1: Table S4). Subsequently, the 72 hypermethylated genes in t(8;21) AML from the DMR7 profile were aligned with the 986 downregulated genes in t(8;21) AML from TCGA, resulting in the identification of 4 key genes, namely, *LIN7A*, *BASP1*, *CEBPA*, and *EMB* (Fig. 2r). Each of these four genes exhibited higher methylation levels in t(8;21) AML and de novo samples than those in non-t(8;21) and CR samples (Fig. 3a, b). Moreover, according to the Meth Primer database, the promoter regions of the four genes were enriched with CpG islands (Fig. 3c). Notably, the expression of *LIN7A* was much lower in patients with t(8;21) translocation than that in patients with other karyotypes (Fig. 3d). Furthermore, patients expressing high *LIN7A* levels showed significantly better survival rates than those expressing low *LIN7A* levels (Fig. 3e).

In addition, to validate the methylation status of the four genes in t(8;21) AML, a publicly available AML methylation dataset (GSE18700) comprising 24 t(8;21) AML and 152 non-t(8;21) AML samples was employed in this study. Through alignment with the 210 hypermethylated DMR7 sequences, a cluster of 10 probes that targeted 7 genes were obtained, among which 3 probes annotated with *LIN7A*, one probe annotated with *BASP1*, and one probe annotated with *EMB* (Fig. 3f). A comparison of the methylation status of these five probes targeted sequences in t(8;21) AML with those in non-t(8;21) AML showed that the *LIN7A*-probe3-targeted sequence was significantly hypermethylated in t(8;21) AML (Fig. 3g). Moreover, t(8;21) patients with a hypermethylated *LIN7A*-probe3-targeted sequence showed poorer overall survival rates (Fig. 3h) and shorter event-free survival than those with a hypomethylated *LIN7A*-probe3-targeted sequence (Fig. 3i). Therefore, we postulate that *LIN7A* may play a significant role in the DAC treatment response in t(8;21) AML.

### Effect of DAC on LIN7A mRNA and protein expression

To determine whether *LIN7A* can serve as a predictor of the t(8;21) AML response to DAC, we conducted an in vitro study exploring the relationship between DAC and *LIN7A* expression. First, we examined whether DAC affects *LIN7A* expression in Kasumi-1 and SKNO-1 cells. Notably, LIN7A expression was significantly elevated upon treatment with 10 nM DAC at the mRNA (Fig. 3j) and protein (Fig. 3k) levels; whereas the expression of DNMT3A, a methyltransferase that could be inhibited by DAC [18], was reduced (Fig. 3k). These results suggest that treatment with DAC promotes the expression of LIN7A in t(8;21) AML cells.

To further elucidate the mechanism of DAC-mediated LIN7A upregulation, we retrieved the DMR sequence mapped to the *LIN7A* promoter region (probe-3 targeted sequence in GSE18700) for methylation-specific PCR analysis. The mapped regions within the *LIN7A* DMR were methylated in the Kasumi-1 cell line (Fig. 3l), suggesting that DAC activates the expression of LIN7A in t(8;21) AML through demethylating of the promoter region.

### Effects of LIN7A downregulation on t(8;21) AML cell death induced by DAC/Ara-C combination therapy

To investigate the role of LIN7A in DAC treatment in t(8;21) AML, we knocked down *LIN7A* in Kasumi-1 and SKNO-1 cells using targeted shRNAs before treatment with DAC (Fig. 3m). We confirmed that *LIN7A* upregulation induced by DAC was counteracted by the presence of sh*LIN7A* in both cell lines (Fig. 3n, o).

**Fig. 4:**
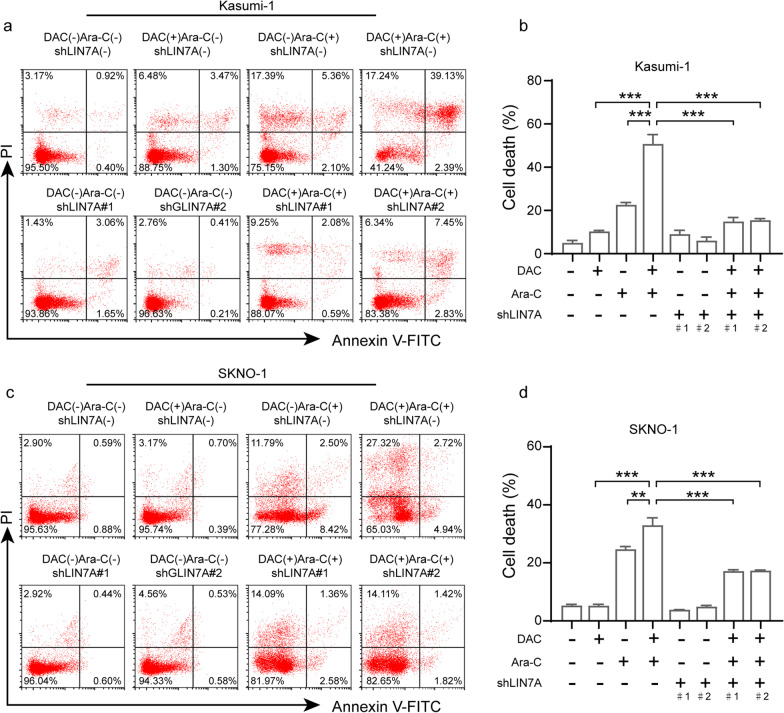
Effect of LIN7A on cell apoptosis undertreatment with DAC in t(8;21) AML cell lines. **a**–**d** Effects of LIN7A on **a**, **b** Kasumi-1 and **c**, **d** SKNO-1 cell apoptosis. Cells were transfected with shRNA lentiviruses and treated with 10 nM DAC and 100 nM Ara-C. Cell apoptosis was assayed using Annexin V-FITC/propidium iodide double staining

Given that the sequential combination of DAC and Ara-C has a synergistic pro-apoptotic effect on AML cells [19], we evaluated the viability of *LIN7A*-knockdown cells after treatment with DAC and Ara-C (Fig. 4a–d). Consistent with previously described findings [8], Kasumi-1 and SKNO-1 cells pre-sensitized with DAC were more sensitive to Ara-C treatment, whereas *LIN7A* knockdown inhibited the synergistic effect of DAC and Ara-C on cell viability. These findings suggest that LIN7A mediates the pre-sensitization effect of DAC on t(8;21) AML cells.

## Discussion

DAC-based regimens are used as an alternative therapy for patients who are ineligible for intensive chemotherapy. However, the clinical efficacy of DAC-based treatments varies greatly among patients, resulting in the currently unmet clinical need for more predictable biomarkers. Genes directly involved in DNA methylation regulation, such as DNA methyltransferases (DNMTs), have been of interest for decades. However, although DNMT1 is directly inhibited by DAC, its expression is unrelated to the DAC response [20]. Moreover, previously confirmed DNA methylation-modified genes (*p15*, *p16*, and *CDH1*) failed to predict DAC efficacy [21]. Notably, a clinical study revealed that patients with t(8;21) AML tend to be more sensitive to DAC-based regimen [8], which may provide new insights into this topic.

Herein, we initially retrieved the DMRs between t(8;21) and non-t (8;21) AML samples. The significant variation observed between AML1-ETO-positive and -negative samples directly indicated the influence of AML1-ETO on the DNA methylation profile. This event may be mediated by DNMT1 [22] and DNMT3A [23], as it has been observed that AML1-ETO can recruit these methyltransferases to DNA. Furthermore, AML1-ETO-positive samples were found to have fewer hypermethylated regions than those of AML1-ETO-negative samples, regardless of whether bone marrow samples or cell lines were employed in the analysis. This may account for why patients with AML1-ETO have a better clinical prognosis. Moreover, given that leukemia blast cells are globally hypermethylated relative to healthy cells, the presence of a hypomethylated profile in AML1-ETO-positive samples may indicate a relatively minor disturbed methylation profile in t(8;21) AML than non-t(8;21) AML.

Although DAC is recognized as a demethylation agent, our study showed that a large number of DNA regions acquired methylation modification following DAC-based combination therapy. This might be a consequence of the combination regimen since eliminating leukemia blast cells drives bone marrow cells to exhibit normal phenotype. However, the methylation restoration effect of DAC cannot be ruled out, considering that numerous DNA regions were methylated in cells treated with DAC only.

Moreover, upon mapping DMRs with reference genes, we observed that more DMRs annotated within the gene body compared with those in promoter regions, thus emphasizing the importance of including non-promoter regions in DNA methylation studies. In contrast to the suppressive effect elicited by methylation in promoter regions, DNA gene body methylation has been reported to promote gene expression [24]. Meanwhile, considering the consistent recognition of the methylation silencing effect of promoter regions, we selected these regions as our subject to develop molecular markers for clinical application. In particular, we found that *LIN7A* may be a key gene that influences DAC treatment efficacy. Furthermore, we demonstrated that LIN7A mediates the synergistic anti-apoptotic effect of DAC and Ara-C treatment in t(8;21) AML.

## Conclusion

Collectively, our study suggests that LIN7A may serve as a specific biomarker for predicting treatment responses to DAC-based therapy in t(8;21) AML patients. However, these findings warrant further confirmation through in vivo studies and clinical assessment.

## Supplementary Information

**Additional file 1: Table S1.** Primers used in MSP analysis. **Table S2.** Primers used in qRT-PCR. **Table S3.** The 72 gene list derived from DMR7. **Table S4.** 986 downregulated genes in t(8;21) AML.

## Data Availability

The methylation sequencing data that support the findings of this study are available in GSE155466, Additional file 1: Tables S3 and S4. The GSE18700 dataset is publicly available in the Gene Expression Omnibus database (https://www.ncbi.nlm.nih.gov/geo/query/acc.cgi?acc=GSE18700).

## Abbreviations

AML: Acute myeloid leukemia
Ara-C: Cytarabine
CR: Complete remission
DAC: Decitabine
DCAG: Decitabine, cytarabine, aclarubicin, and granulocyte colony-stimulating factor
DMR: Differentially methylated region
DNMT: DNA methyltransferase
PCR: Polymerase chain reaction
qRT-PCR: Quantitative real-time PCR
shRNA: Short hairpin RNA
TSS: Transcription start site

## References

[1] Meyer T, Jahn N, Lindner S, Röhner L, Dolnik A, Weber D (2020). Functional characterization of BRCC3 mutations in acute myeloid leukemia with t(8;21)(q22;q22.1). Leukemia.

[2] Ganetsky A (2012). The role of decitabine for the treatment of acute myeloid leukemia. Ann Pharmacother.

[3] Khan N, Hantel A, Knoebel RW, Artz A, Larson RA, Godley LA (2017). Efficacy of single-agent decitabine in relapsed and refractory acute myeloid leukemia. Leuk Lymphoma.

[4] Issa JP, Garcia-Manero G, Giles FJ, Mannari R, Thomas D, Faderl S (2004). Phase 1 study of low-dose prolonged exposure schedules of the hypomethylating agent 5-aza-2′-deoxycytidine (decitabine) in hematopoietic malignancies. Blood.

[5] Kantarjian HM, Thomas XG, Dmoszynska A, Wierzbowska A, Mazur G, Mayer J (2012). Multicenter, randomized, open-label, phase III trial of decitabine versus patient choice, with physician advice, of either supportive care or low-dose cytarabine for the treatment of older patients with newly diagnosed acute myeloid leukemia. J Clin Oncol Off J Am Soc Clin Oncol.

[6] Pollyea DA, Pratz K, Letai A, Jonas BA, Wei AH, Pullarkat V (2021). Venetoclax with azacitidine or decitabine in patients with newly diagnosed acute myeloid leukemia: long term follow-up from a phase 1b study. Am J Hematol.

[7] Jonas BA, Pollyea DA (2019). How we use venetoclax with hypomethylating agents for the treatment of newly diagnosed patients with acute myeloid leukemia. Leukemia.

[8] Wang L, Luo J, Chen G, Fang M, Wei X, Li Y (2020). Chidamide, decitabine, cytarabine, aclarubicin, and granulocyte colony-stimulating factor (CDCAG) in patients with relapsed/refractory acute myeloid leukemia: a single-arm, phase 1/2 study. Clin Epigenetics.

[9] Liu N, Song J, Xie Y, Wang XL, Rong B, Man N (2019). Different roles of E proteins in t(8;21) leukemia: E2–2 compromises the function of AETFC and negatively regulates leukemogenesis. Proc Natl Acad Sci USA.

[10] Xu Y, Man N, Karl D, Martinez C, Liu F, Sun J (2019). TAF1 plays a critical role in AML1-ETO driven leukemogenesis. Nat Commun.

[11] Li Y, Ning Q, Shi J, Chen Y, Jiang M, Gao L (2017). A novel epigenetic AML1-ETO/THAP10/miR-383 mini-circuitry contributes to t(8;21) leukaemogenesis. EMBO Mol Med.

[12] Figueroa ME, Lugthart S, Li Y, Erpelinck-Verschueren C, Deng X, Christos PJ (2010). DNA methylation signatures identify biologically distinct subtypes in acute myeloid leukemia. Cancer Cell.

[13] Li Y, Zhao H, Xu Q, Lv N, Jing Y, Wang L (2017). Detection of prognostic methylation markers by methylC-capture sequencing in acute myeloid leukemia. Oncotarget.

[14] Kunde-Ramamoorthy G, Coarfa C, Laritsky E, Kessler NJ, Harris RA, Xu M (2014). Comparison and quantitative verification of mapping algorithms for whole-genome bisulfite sequencing. Nucleic Acids Res.

[15] Quinlan AR, Hall IM (2010). BEDTools: a flexible suite of utilities for comparing genomic features. Bioinform (Oxford, England).

[16] Wang K, Li M, Hakonarson H (2010). ANNOVAR: functional annotation of genetic variants from high-throughput sequencing data. Nucleic Acids Res.

[17] Yang E, Guan W, Gong D, Gao X, Han C, Zhang J (2020). Epigenetic silencing of miR564 contributes to the leukemogenesis of t(8;21) acute myeloid leukemia. Clin Sci (London, England: 1979).

[18] Pappalardi MB, Keenan K, Cockerill M, Kellner WA, Stowell A, Sherk C (2021). Discovery of a first-in-class reversible DNMT1-selective inhibitor with improved tolerability and efficacy in acute myeloid leukemia. Nature cancer.

[19] Qin T, Youssef EM, Jelinek J, Chen R, Yang AS, Garcia-Manero G (2007). Effect of cytarabine and decitabine in combination in human leukemic cell lines. Clin Cancer Res.

[20] Blum W, Klisovic RB, Hackanson B, Liu Z, Liu S, Devine H (2007). Phase I study of decitabine alone or in combination with valproic acid in acute myeloid leukemia. J Clin Oncol Off J Am Soc Clin Oncol.

[21] Daskalakis M, Nguyen TT, Nguyen C, Guldberg P, Köhler G, Wijermans P (2002). Demethylation of a hypermethylated P15/INK4B gene in patients with myelodysplastic syndrome by 5-Aza-2′-deoxycytidine (decitabine) treatment. Blood.

[22] Liu S, Shen T, Huynh L, Klisovic MI, Rush LJ, Ford JL (2005). Interplay of RUNX1/MTG8 and DNA methyltransferase 1 in acute myeloid leukemia. Can Res.

[23] Zhou L, Fu L, Lv N, Liu J, Li Y, Chen X (2018). Methylation-associated silencing of BASP1 contributes to leukemogenesis in t(8;21) acute myeloid leukemia. Exp Mol Med.

[24] Yang X, Han H, De Carvalho DD, Lay FD, Jones PA, Liang G (2014). Gene body methylation can alter gene expression and is a therapeutic target in cancer. Cancer Cell.

