# Supplementary material for: DNA methylation landscape reveals LIN7A as a decitabine-responsive marker in patients with t(8;21) acute myeloid leukemia
Source: Clin Epigenetics. 2023 Mar 3;15:37. doi: 10.1186/s13148-023-01458-0 (PMC9983225; doi:10.1186/s13148-023-01458-0)
Supplement: Supplementary file 1 — Additional file 1: Table S1. Primers used in MSP analysis. Table S2. Primers used in qRT-PCR. Table S3. The 72 gene list derived from DMR7. Table S4. 986 downregulated genes in t(8;21) AML. [file 13148_2023_1458_MOESM1_ESM.docx]

Additional table S1. Primers used in MSP analysis

| Primer 1 | Methylated | Forward | TGCGTTAAGTAAGTTGATAGGC |
| --- | --- | --- | --- |
|  |  | Reverse | TTTATTAAAACCGCTACGCGA |
|  | Un-methylated | Forward | GTGTTAAGTAAGTTGATAGGTGG |
|  |  | Reverse | TCTAACTTTATTAAAACCACTACACAAAA |
| Primer 2 | Methylated | Forward | GCGTTAAGTAAGTTGATAGGCG |
|  |  | Reverse | TTATTAAAACCGCTACGCGA |
|  | Un-methylated | Forward | GTGTTAAGTAAGTTGATAGGTGG |
|  |  | Reverse | TCTAACTTTATTAAAACCACTACACAAAA |

Additional Table S2. Primers used in qRT-PCR

| LIN7A | Forward | ACCCTGGACAGAGGTGTATCA |
| --- | --- | --- |
|  | Reverse | ACTGTTGCCTTTGCTGTTGC |
| GAPDH | Forward | CTCTGCTCCTCCTGTTCGAC |
|  | Reverse | GCCCAATACGACCAAATCC |

Additional Table S3. The 72 gene list derived from DMR7

| Number | Chromosome | Start | End | Gene | Region |
| --- | --- | --- | --- | --- | --- |
| 1 | chr1 | 60392391 | 60392477 | CYP2J2 | promoter |
| 2 | chr1 | 1.04E+08 | 1.04E+08 | COL11A1 | promoter |
| 3 | chr1 | 1.53E+08 | 1.53E+08 | LCE3A | promoter |
| 4 | chr1 | 1.59E+08 | 1.59E+08 | AIM2 | promoter |
| 5 | chr1 | 2E+08 | 2E+08 | NR5A2 | promoter |
| 6 | chr1 | 2.34E+08 | 2.34E+08 | KCNK1 | promoter |
| 7 | chr10 | 50818945 | 50819047 | CHAT | promoter |
| 8 | chr11 | 6947501 | 6947584 | ZNF215 | promoter |
| 9 | chr11 | 26353570 | 26353575 | ANO3 | promoter |
| 9 | chr11 | 26353584 | 26353619 | ANO3 | promoter |
| 10 | chr11 | 1.01E+08 | 1.01E+08 | LOC101054525 | promoter |
| 10 | chr11 | 1.01E+08 | 1.01E+08 | LOC101054525 | promoter |
| 11 | chr11 | 1.01E+08 | 1.01E+08 | PGR | promoter |
| 12 | chr11 | 1.27E+08 | 1.27E+08 | KIRREL3 | promoter |
| 13 | chr11 | 1.32E+08 | 1.32E+08 | NTM | promoter |
| 14 | chr12 | 5541252 | 5541270 | NTF3 | promoter |
| 15 | chr12 | 16761405 | 16761421 | LMO3 | promoter |
| 16 | chr12 | 29937310 | 29937358 | TMTC1 | promoter |
| 17 | chr12 | 45444962 | 45445203 | DBX2 | promoter |
| 18 | chr12 | 81331646 | 81331722 | LIN7A | promoter |
| 19 | chr12 | 81331646 | 81331722 | MIR618 | promoter |
| 20 | chr12 | 1.17E+08 | 1.17E+08 | C12orf49 | promoter |
| 21 | chr12 | 1.17E+08 | 1.17E+08 | RNFT2 | promoter |
| 22 | chr12 | 1.19E+08 | 1.19E+08 | SRRM4 | promoter |
| 23 | chr13 | 96705708 | 96705821 | UGGT2 | promoter |
| 24 | chr15 | 53083654 | 53083978 | ONECUT1 | promoter |
| 25 | chr15 | 96875526 | 96875596 | MIR1469 | promoter |
| 26 | chr15 | 96875526 | 96875596 | NR2F2 | promoter |
| 25 | chr15 | 96875651 | 96875675 | MIR1469 | promoter |
| 26 | chr15 | 96875651 | 96875675 | NR2F2 | promoter |
| 25 | chr15 | 96875688 | 96875716 | MIR1469 | promoter |
| 26 | chr15 | 96875688 | 96875716 | NR2F2 | promoter |
| 25 | chr15 | 96876250 | 96876265 | MIR1469 | promoter |
| 26 | chr15 | 96876250 | 96876265 | NR2F2 | promoter |
| 25 | chr15 | 96876452 | 96876678 | MIR1469 | promoter |
| 26 | chr15 | 96876452 | 96876678 | NR2F2 | promoter |
| 27 | chr17 | 46711277 | 46711370 | MIR196A1 | promoter |
| 28 | chr17 | 47072902 | 47072994 | IGF2BP1 | promoter |
| 29 | chr18 | 5543772 | 5543986 | EPB41L3 | promoter |
| 30 | chr18 | 40857237 | 40857435 | SYT4 | promoter |
| 31 | chr19 | 9608805 | 9608998 | ZNF560 | promoter |
| 32 | chr19 | 33795318 | 33795513 | CEBPA | promoter |
| 33 | chr19 | 52390788 | 52391440 | ZNF577 | promoter |
| 34 | chr19 | 52390788 | 52391440 | ZNF649-AS1 | promoter |
| 35 | chr2 | 1.31E+08 | 1.31E+08 | RAB6C-AS1 | promoter |
| 36 | chr2 | 2.2E+08 | 2.2E+08 | CFAP65 | promoter |
| 37 | chr3 | 13974650 | 13974741 | FGD5P1 | promoter |
| 38 | chr3 | 48700288 | 48700375 | CELSR3-AS1 | promoter |
| 39 | chr3 | 48700288 | 48700375 | CELSR3 | promoter |
| 40 | chr3 | 64674245 | 64674467 | ADAMTS9 | promoter |
| 41 | chr3 | 1.08E+08 | 1.08E+08 | HHLA2 | promoter |
| 42 | chr3 | 1.16E+08 | 1.16E+08 | LSAMP | promoter |
| 43 | chr3 | 1.69E+08 | 1.69E+08 | MECOM | promoter |
| 44 | chr3 | 1.73E+08 | 1.73E+08 | NLGN1 | promoter |
| 45 | chr3 | 1.8E+08 | 1.8E+08 | CCDC39 | promoter |
| 46 | chr3 | 1.85E+08 | 1.85E+08 | MAP3K13 | promoter |
| 47 | chr3 | 1.92E+08 | 1.92E+08 | FGF12 | promoter |
| 48 | chr4 | 44451497 | 44451533 | KCTD8 | promoter |
| 49 | chr4 | 46392368 | 46393033 | GABRA2 | promoter |
| 50 | chr4 | 1.86E+08 | 1.86E+08 | ANKRD37 | promoter |
| 51 | chr5 | 3594243 | 3594278 | IRX1 | promoter |
| 52 | chr5 | 17216993 | 17217036 | BASP1 | promoter |
| 53 | chr5 | 35230401 | 35230530 | PRLR | promoter |
| 54 | chr5 | 49739093 | 49739326 | EMB | promoter |
| 55 | chr5 | 50678361 | 50678477 | ISL1 | promoter |
| 56 | chr6 | 6004896 | 6004930 | NRN1 | promoter |
| 56 | chr6 | 6004932 | 6004948 | NRN1 | promoter |
| 57 | chr6 | 17101782 | 17101943 | STMND1 | promoter |
| 58 | chr6 | 46139119 | 46139152 | ENPP5 | promoter |
| 59 | chr6 | 1.17E+08 | 1.17E+08 | DSE | promoter |
| 60 | chr6 | 1.5E+08 | 1.5E+08 | ULBP1 | promoter |
| 61 | chr6 | 1.53E+08 | 1.53E+08 | SYNE1 | promoter |
| 62 | chr7 | 27225722 | 27225772 | HOXA11 | promoter |
| 63 | chr7 | 28450620 | 28450747 | CREB5 | promoter |
| 64 | chr7 | 86850311 | 86850376 | TMEM243 | promoter |
| 65 | chr7 | 88389046 | 88389079 | ZNF804B | promoter |
| 66 | chr7 | 89874234 | 89874257 | CFAP69 | promoter |
| 67 | chr7 | 1.31E+08 | 1.31E+08 | LINC-PINT | promoter |
| 68 | chr7 | 1.31E+08 | 1.31E+08 | MKLN1 | promoter |
| 69 | chr8 | 9762248 | 9762289 | LINC00599 | promoter |
| 70 | chr8 | 9762248 | 9762289 | MIR124-1 | promoter |
| 71 | chr8 | 79577781 | 79578036 | ZC2HC1A | promoter |
| 71 | chr8 | 79578112 | 79578144 | ZC2HC1A | promoter |
| 71 | chr8 | 79578149 | 79578437 | ZC2HC1A | promoter |
| 72 | chr8 | 89340125 | 89340188 | MMP16 | promoter |

Additional Table S4. 986 downregulated genes in t(8;21) AML

| No. | Gene | Relative Expression level in  t(8;21) AML | Relative Expression level in  non-t(8;21) AML | logFC | pValue | FDR |
| --- | --- | --- | --- | --- | --- | --- |
| 1 | IFT57 | 3.48 | 6.89 | 3.40 | 1.42E-03 | 0.02 |
| 2 | AC035139.1 | 1.16 | 4.66 | 3.50 | 3.60E-04 | 0.01 |
| 3 | PI4KA | 16.44 | 27.37 | 10.92 | 2.36E-04 | 0.01 |
| 4 | SSX2IP | 1.09 | 4.16 | 3.06 | 2.12E-05 | 0.00 |
| 5 | IGHD1-7 | 0.31 | 50.64 | 50.33 | 8.54E-05 | 0.00 |
| 6 | LILRB1 | 0.94 | 8.25 | 7.31 | 7.28E-04 | 0.01 |
| 7 | RN7SL357P | 0.04 | 3.32 | 3.28 | 3.04E-04 | 0.01 |
| 8 | SLC9A8 | 7.20 | 9.70 | 2.50 | 5.38E-03 | 0.04 |
| 9 | PDK3 | 4.45 | 7.77 | 3.31 | 2.19E-04 | 0.01 |
| 10 | PITPNA | 12.96 | 17.43 | 4.47 | 1.99E-03 | 0.02 |
| 11 | SLC35F6 | 4.48 | 8.29 | 3.81 | 3.70E-03 | 0.03 |
| 12 | IGHD3-10 | 0.89 | 45.89 | 45.00 | 1.49E-04 | 0.01 |
| 13 | TJP2 | 1.77 | 4.66 | 2.90 | 6.45E-03 | 0.05 |
| 14 | PARVG | 12.61 | 26.72 | 14.10 | 1.66E-04 | 0.01 |
| 15 | PPBP | 1.50 | 38.96 | 37.46 | 2.77E-03 | 0.03 |
| 16 | FCN1 | 1.96 | 61.31 | 59.35 | 4.04E-04 | 0.01 |
| 17 | CSGALNACT2 | 16.80 | 29.49 | 12.69 | 7.02E-04 | 0.01 |
| 18 | NXPE3 | 3.92 | 7.25 | 3.33 | 1.99E-03 | 0.02 |
| 19 | RBM38 | 4.73 | 20.46 | 15.73 | 6.22E-05 | 0.00 |
| 20 | TNS3 | 4.25 | 12.89 | 8.64 | 1.57E-03 | 0.02 |
| 21 | CLEC2D | 2.89 | 9.64 | 6.75 | 4.83E-05 | 0.00 |
| 22 | PRKAR2B | 2.20 | 13.38 | 11.19 | 3.47E-04 | 0.01 |
| 23 | TRAF3IP3 | 9.26 | 16.28 | 7.01 | 2.20E-03 | 0.02 |
| 24 | IFI30 | 0.34 | 2.76 | 2.42 | 4.61E-03 | 0.04 |
| 25 | SLC36A1 | 3.67 | 10.65 | 6.98 | 9.68E-04 | 0.02 |
| 26 | PADI2 | 0.32 | 4.72 | 4.40 | 7.02E-04 | 0.01 |
| 27 | RN7SL368P | 1.43 | 11.62 | 10.20 | 2.36E-04 | 0.01 |
| 28 | VPS26B | 10.50 | 14.23 | 3.73 | 4.19E-04 | 0.01 |
| 29 | AL109914.1 | 0.38 | 5.48 | 5.11 | 8.37E-04 | 0.01 |
| 30 | SYK | 28.40 | 58.95 | 30.55 | 7.67E-05 | 0.00 |
| 31 | SH3BP5-AS1 | 2.74 | 5.49 | 2.75 | 1.86E-03 | 0.02 |
| 32 | TM4SF1 | 0.12 | 4.32 | 4.20 | 1.24E-03 | 0.02 |
| 33 | SERINC2 | 0.25 | 2.86 | 2.61 | 5.21E-03 | 0.04 |
| 34 | RNF166 | 8.49 | 20.84 | 12.34 | 7.99E-05 | 0.00 |
| 35 | PRKAG1 | 7.28 | 9.40 | 2.12 | 4.20E-03 | 0.04 |
| 36 | AC090559.1 | 21.13 | 42.84 | 21.71 | 2.96E-03 | 0.03 |
| 37 | ATXN1 | 0.57 | 3.41 | 2.84 | 1.66E-04 | 0.01 |
| 38 | CCDC102A | 0.61 | 2.70 | 2.09 | 3.34E-04 | 0.01 |
| 39 | KLHDC8B | 0.61 | 3.16 | 2.55 | 6.77E-04 | 0.01 |
| 40 | ARID5A | 5.95 | 12.72 | 6.77 | 3.89E-04 | 0.01 |
| 41 | CSK | 25.09 | 37.84 | 12.75 | 3.94E-03 | 0.04 |
| 42 | VOPP1 | 0.58 | 3.64 | 3.06 | 1.35E-05 | 0.00 |
| 43 | SLC49A3 | 0.99 | 3.20 | 2.22 | 3.94E-03 | 0.04 |
| 44 | MPEG1 | 2.54 | 92.77 | 90.23 | 2.10E-04 | 0.01 |
| 45 | AC016292.1 | 0.74 | 6.28 | 5.54 | 2.10E-04 | 0.01 |
| 46 | IGHD5-5 | 0.43 | 66.50 | 66.06 | 6.15E-05 | 0.00 |
| 47 | VAMP2 | 18.60 | 30.02 | 11.43 | 8.40E-04 | 0.01 |
| 48 | PRKD2 | 7.18 | 13.01 | 5.83 | 3.58E-03 | 0.03 |
| 49 | TLR7 | 0.37 | 3.83 | 3.47 | 5.71E-03 | 0.04 |
| 50 | GOLGA8B | 1.08 | 6.88 | 5.81 | 1.04E-03 | 0.02 |
| 51 | KLHL2 | 3.88 | 8.04 | 4.16 | 6.77E-04 | 0.01 |
| 52 | DPYSL3 | 0.22 | 2.79 | 2.57 | 6.26E-03 | 0.05 |
| 53 | ITGA7 | 0.33 | 2.87 | 2.54 | 3.34E-04 | 0.01 |
| 54 | TGFB1 | 41.72 | 94.27 | 52.54 | 3.34E-04 | 0.01 |
| 55 | NOTCH2 | 8.65 | 23.56 | 14.91 | 1.47E-04 | 0.01 |
| 56 | ALOX5AP | 3.98 | 38.68 | 34.70 | 1.30E-04 | 0.01 |
| 57 | RNF19B | 6.89 | 12.69 | 5.80 | 4.69E-04 | 0.01 |
| 58 | KRI1 | 9.58 | 17.93 | 8.35 | 5.97E-05 | 0.00 |
| 59 | PTPRE | 9.03 | 19.63 | 10.59 | 6.65E-03 | 0.05 |
| 60 | RNU6-1280P | 4.99 | 12.80 | 7.81 | 1.93E-03 | 0.02 |
| 61 | CHST2 | 1.42 | 5.54 | 4.12 | 1.28E-03 | 0.02 |
| 62 | CNPY3 | 72.68 | 104.73 | 32.05 | 5.38E-03 | 0.04 |
| 63 | AC005264.1 | 3.85 | 12.32 | 8.48 | 2.86E-03 | 0.03 |
| 64 | ITGB2-AS1 | 1.39 | 11.72 | 10.32 | 1.16E-04 | 0.01 |
| 65 | RFLNB | 17.53 | 65.89 | 48.36 | 4.63E-05 | 0.00 |
| 66 | AL023653.1 | 1.67 | 6.14 | 4.46 | 6.77E-05 | 0.00 |
| 67 | MIR4298 | 1.04 | 7.67 | 6.64 | 3.28E-04 | 0.01 |
| 68 | GRN | 36.77 | 161.91 | 125.14 | 1.08E-03 | 0.02 |
| 69 | PLXNB2 | 24.43 | 58.61 | 34.18 | 4.52E-04 | 0.01 |
| 70 | AC116348.2 | 0.90 | 3.26 | 2.36 | 2.36E-04 | 0.01 |
| 71 | HOPX | 0.77 | 4.94 | 4.17 | 6.85E-03 | 0.05 |
| 72 | AC099494.2 | 1.12 | 4.42 | 3.30 | 3.74E-05 | 0.00 |
| 73 | MR1 | 5.33 | 7.55 | 2.22 | 3.26E-03 | 0.03 |
| 74 | ALDH1A1 | 0.30 | 6.91 | 6.61 | 3.94E-03 | 0.04 |
| 75 | WWP1 | 5.46 | 7.71 | 2.25 | 5.89E-03 | 0.04 |
| 76 | CPEB4 | 3.46 | 6.73 | 3.27 | 4.20E-03 | 0.04 |
| 77 | SHTN1 | 0.04 | 2.17 | 2.13 | 5.04E-05 | 0.00 |
| 78 | NLRP12 | 0.23 | 6.33 | 6.10 | 9.82E-05 | 0.01 |
| 79 | RNF24 | 28.13 | 41.66 | 13.53 | 5.54E-03 | 0.04 |
| 80 | ATP1B1 | 2.51 | 8.61 | 6.10 | 1.15E-03 | 0.02 |
| 81 | ADGRG5 | 1.02 | 14.52 | 13.50 | 3.01E-05 | 0.00 |
| 82 | AC004593.1 | 0.17 | 2.57 | 2.40 | 1.30E-04 | 0.01 |
| 83 | RABGAP1 | 5.08 | 8.04 | 2.95 | 2.28E-03 | 0.03 |
| 84 | OSBPL11 | 4.40 | 9.33 | 4.93 | 1.33E-03 | 0.02 |
| 85 | AC099524.1 | 0.30 | 2.31 | 2.01 | 3.01E-05 | 0.00 |
| 86 | ERO1A | 8.44 | 12.69 | 4.25 | 5.06E-03 | 0.04 |
| 87 | GPAT3 | 0.21 | 5.40 | 5.19 | 1.02E-04 | 0.01 |
| 88 | LGALS8 | 10.60 | 14.66 | 4.05 | 2.96E-03 | 0.03 |
| 89 | AC068580.1 | 0.62 | 3.26 | 2.64 | 1.16E-04 | 0.01 |
| 90 | RPS2P36 | 1.76 | 4.91 | 3.15 | 2.10E-04 | 0.01 |
| 91 | ANXA4 | 3.55 | 5.82 | 2.27 | 1.11E-03 | 0.02 |
| 92 | CLEC7A | 1.13 | 13.65 | 12.51 | 2.13E-03 | 0.02 |
| 93 | HOXA9 | 0.58 | 22.10 | 21.52 | 5.64E-04 | 0.01 |
| 94 | FAM53B | 7.54 | 11.72 | 4.18 | 1.74E-03 | 0.02 |
| 95 | CSTA | 3.75 | 27.40 | 23.65 | 3.26E-03 | 0.03 |
| 96 | NPRL2 | 5.30 | 7.87 | 2.57 | 4.90E-03 | 0.04 |
| 97 | NFKBID | 8.18 | 15.99 | 7.82 | 3.70E-03 | 0.03 |
| 98 | WDR1 | 25.77 | 34.85 | 9.08 | 1.42E-03 | 0.02 |
| 99 | FAM219A | 2.88 | 5.18 | 2.30 | 1.00E-03 | 0.02 |
| 100 | SRGN | 311.20 | 1556.72 | 1245.51 | 1.86E-03 | 0.02 |
| 101 | NEGR1 | 0.08 | 4.21 | 4.13 | 9.43E-05 | 0.00 |
| 102 | EMILIN1 | 0.63 | 6.67 | 6.04 | 3.26E-03 | 0.03 |
| 103 | SRC | 0.71 | 4.48 | 3.77 | 1.87E-04 | 0.01 |
| 104 | CCL3 | 0.64 | 2.96 | 2.32 | 2.20E-03 | 0.02 |
| 105 | FGD2 | 1.03 | 8.75 | 7.72 | 9.68E-04 | 0.02 |
| 106 | PAK1 | 9.48 | 21.48 | 12.00 | 1.33E-03 | 0.02 |
| 107 | RNF168 | 7.62 | 10.61 | 2.99 | 2.96E-03 | 0.03 |
| 108 | ITGAL | 15.41 | 57.29 | 41.89 | 1.94E-04 | 0.01 |
| 109 | NFE2 | 36.43 | 83.43 | 47.00 | 6.07E-04 | 0.01 |
| 110 | ELL2 | 1.48 | 5.03 | 3.55 | 1.47E-03 | 0.02 |
| 111 | AIF1 | 31.17 | 105.08 | 73.91 | 1.53E-04 | 0.01 |
| 112 | AMPD3 | 3.91 | 8.41 | 4.50 | 2.10E-04 | 0.01 |
| 113 | PDLIM5 | 2.66 | 5.00 | 2.35 | 6.85E-03 | 0.05 |
| 114 | ATP2B4 | 8.60 | 36.64 | 28.04 | 1.13E-05 | 0.00 |
| 115 | HOXB5 | 0.03 | 4.74 | 4.71 | 2.46E-03 | 0.03 |
| 116 | ALCAM | 3.69 | 12.24 | 8.54 | 1.20E-04 | 0.01 |
| 117 | TMEM150B | 0.66 | 4.96 | 4.30 | 1.15E-03 | 0.02 |
| 118 | AC003070.1 | 4.75 | 10.58 | 5.83 | 4.52E-04 | 0.01 |
| 119 | FAM129B | 0.64 | 7.90 | 7.26 | 1.07E-04 | 0.01 |
| 120 | CREG1 | 50.10 | 74.26 | 24.16 | 5.38E-03 | 0.04 |
| 121 | PIK3R5 | 6.71 | 13.60 | 6.89 | 2.28E-03 | 0.03 |
| 122 | NEK9 | 10.28 | 13.53 | 3.24 | 2.20E-03 | 0.02 |
| 123 | MIR3183 | 0.94 | 3.00 | 2.07 | 6.74E-03 | 0.05 |
| 124 | MIR6774 | 1.46 | 11.92 | 10.46 | 2.51E-03 | 0.03 |
| 125 | AL365318.1 | 0.18 | 2.55 | 2.37 | 4.33E-03 | 0.04 |
| 126 | ATP5MC2P3 | 3.67 | 7.45 | 3.78 | 2.51E-03 | 0.03 |
| 127 | CHMP1B | 16.51 | 20.89 | 4.38 | 5.21E-03 | 0.04 |
| 128 | HOXB6 | 0.02 | 5.25 | 5.23 | 7.01E-04 | 0.01 |
| 129 | MYH9 | 91.91 | 143.30 | 51.39 | 3.82E-03 | 0.03 |
| 130 | GABBR1 | 0.96 | 7.91 | 6.95 | 4.90E-03 | 0.04 |
| 131 | AC017035.1 | 1.33 | 3.60 | 2.26 | 2.36E-04 | 0.01 |
| 132 | RMND5B | 1.68 | 4.92 | 3.24 | 4.63E-05 | 0.00 |
| 133 | TRAM2 | 6.85 | 10.66 | 3.82 | 2.20E-03 | 0.02 |
| 134 | AL683807.1 | 1.55 | 6.87 | 5.32 | 2.35E-03 | 0.03 |
| 135 | VIM | 100.78 | 331.93 | 231.15 | 7.82E-04 | 0.01 |
| 136 | CLN3 | 3.86 | 6.05 | 2.18 | 2.60E-03 | 0.03 |
| 137 | HSPE1P18 | 0.49 | 4.95 | 4.46 | 3.26E-03 | 0.03 |
| 138 | STX12 | 8.85 | 12.20 | 3.35 | 5.71E-03 | 0.04 |
| 139 | KCNK17 | 0.29 | 8.21 | 7.91 | 1.59E-04 | 0.01 |
| 140 | AC092868.1 | 0.19 | 3.01 | 2.81 | 1.04E-03 | 0.02 |
| 141 | MAP3K5 | 9.21 | 17.59 | 8.38 | 7.02E-04 | 0.01 |
| 142 | FMNL3 | 1.45 | 4.00 | 2.55 | 2.68E-03 | 0.03 |
| 143 | SPON2 | 1.95 | 4.73 | 2.78 | 5.54E-03 | 0.04 |
| 144 | JAZF1 | 1.02 | 4.99 | 3.98 | 1.20E-04 | 0.01 |
| 145 | PLXNC1 | 1.96 | 11.25 | 9.30 | 9.05E-05 | 0.00 |
| 146 | RASA3 | 8.62 | 15.98 | 7.36 | 2.77E-03 | 0.03 |
| 147 | MVB12B | 1.45 | 4.24 | 2.79 | 1.11E-03 | 0.02 |
| 148 | TK2 | 1.90 | 4.55 | 2.65 | 7.99E-05 | 0.00 |
| 149 | LTB4R | 8.32 | 21.66 | 13.34 | 9.43E-05 | 0.00 |
| 150 | SLC15A3 | 0.27 | 3.82 | 3.56 | 1.94E-04 | 0.01 |
| 151 | KAT2B | 6.25 | 11.04 | 4.79 | 9.02E-04 | 0.01 |
| 152 | AC006946.1 | 2.35 | 9.66 | 7.30 | 1.80E-03 | 0.02 |
| 153 | CBX6 | 3.06 | 9.45 | 6.39 | 7.06E-05 | 0.00 |
| 154 | IGHD5-18 | 0.23 | 57.63 | 57.40 | 9.60E-05 | 0.01 |
| 155 | IGHD1-1 | 1.31 | 68.45 | 67.14 | 1.22E-04 | 0.01 |
| 156 | FRY | 3.36 | 8.05 | 4.69 | 4.07E-03 | 0.04 |
| 157 | PLAUR | 3.70 | 12.33 | 8.62 | 1.37E-03 | 0.02 |
| 158 | TBC1D8 | 1.50 | 6.16 | 4.66 | 6.49E-05 | 0.00 |
| 159 | ARHGAP21 | 0.60 | 4.57 | 3.97 | 5.43E-04 | 0.01 |
| 160 | GCNT1 | 3.55 | 10.33 | 6.78 | 7.36E-05 | 0.00 |
| 161 | DOP1A | 3.38 | 5.98 | 2.60 | 1.24E-03 | 0.02 |
| 162 | CRIM1 | 1.71 | 4.78 | 3.06 | 3.15E-03 | 0.03 |
| 163 | PANX2 | 0.11 | 2.46 | 2.34 | 1.62E-05 | 0.00 |
| 164 | VAV1 | 14.27 | 28.41 | 14.14 | 2.31E-05 | 0.00 |
| 165 | FGR | 5.23 | 63.87 | 58.64 | 8.33E-05 | 0.00 |
| 166 | PHOSPHO1 | 0.55 | 3.25 | 2.71 | 1.15E-03 | 0.02 |
| 167 | SLC39A11 | 5.75 | 9.14 | 3.39 | 6.85E-03 | 0.05 |
| 168 | BTG2 | 17.89 | 47.90 | 30.01 | 1.24E-03 | 0.02 |
| 169 | RNU7-195P | 0.76 | 3.26 | 2.50 | 1.57E-03 | 0.02 |
| 170 | S100A4 | 65.40 | 157.39 | 91.99 | 3.47E-03 | 0.03 |
| 171 | CALCRL | 0.46 | 3.81 | 3.35 | 6.85E-03 | 0.05 |
| 172 | ITGAM | 3.75 | 32.55 | 28.80 | 1.11E-04 | 0.01 |
| 173 | AL035587.1 | 8.46 | 12.08 | 3.62 | 1.42E-03 | 0.02 |
| 174 | LPXN | 13.86 | 27.66 | 13.80 | 3.82E-03 | 0.03 |
| 175 | CARD16 | 2.60 | 7.55 | 4.95 | 1.16E-04 | 0.01 |
| 176 | MEIS1 | 0.02 | 9.73 | 9.71 | 3.14E-05 | 0.00 |
| 177 | HOXA7 | 0.00 | 2.91 | 2.91 | 5.22E-05 | 0.00 |
| 178 | SCIMP | 0.97 | 10.03 | 9.06 | 3.22E-04 | 0.01 |
| 179 | MIR22HG | 1.25 | 5.83 | 4.58 | 7.54E-04 | 0.01 |
| 180 | SAT2 | 5.68 | 9.42 | 3.73 | 6.85E-03 | 0.05 |
| 181 | PLPPR2 | 4.85 | 11.17 | 6.31 | 3.82E-03 | 0.03 |
| 182 | UBP1 | 11.00 | 13.50 | 2.50 | 4.33E-03 | 0.04 |
| 183 | SLC31A1 | 3.55 | 6.51 | 2.96 | 5.06E-03 | 0.04 |
| 184 | PLBD2 | 3.68 | 13.84 | 10.16 | 3.58E-05 | 0.00 |
| 185 | CTSW | 5.83 | 74.54 | 68.71 | 2.31E-05 | 0.00 |
| 186 | AC099494.1 | 0.82 | 3.49 | 2.66 | 4.07E-05 | 0.00 |
| 187 | TLN1 | 63.31 | 102.32 | 39.01 | 6.07E-03 | 0.05 |
| 188 | AC003102.1 | 1.43 | 3.53 | 2.11 | 2.55E-04 | 0.01 |
| 189 | CD300E | 0.40 | 20.73 | 20.33 | 1.99E-03 | 0.02 |
| 190 | CAST | 6.09 | 14.00 | 7.91 | 6.85E-03 | 0.05 |
| 191 | SUSD1 | 10.15 | 20.03 | 9.88 | 1.66E-04 | 0.01 |
| 192 | PLEKHM1 | 5.95 | 9.09 | 3.14 | 9.68E-04 | 0.02 |
| 193 | CCDC6 | 8.77 | 12.82 | 4.05 | 3.15E-03 | 0.03 |
| 194 | FRMD4B | 1.75 | 4.71 | 2.96 | 4.90E-03 | 0.04 |
| 195 | ADAMTS14 | 0.22 | 2.37 | 2.14 | 3.47E-03 | 0.03 |
| 196 | PEA15 | 9.62 | 20.17 | 10.56 | 2.65E-04 | 0.01 |
| 197 | FAM30A | 0.58 | 37.62 | 37.04 | 3.14E-05 | 0.00 |
| 198 | DPYD | 6.45 | 15.77 | 9.32 | 5.54E-03 | 0.04 |
| 199 | JAM3 | 0.75 | 2.79 | 2.04 | 2.19E-04 | 0.01 |
| 200 | RAPGEF2 | 4.08 | 8.18 | 4.09 | 2.51E-03 | 0.03 |
| 201 | AD000864.1 | 1.87 | 4.38 | 2.51 | 1.42E-03 | 0.02 |
| 202 | PIK3AP1 | 19.76 | 38.40 | 18.65 | 1.08E-03 | 0.02 |
| 203 | XYLT1 | 5.43 | 13.11 | 7.68 | 1.04E-03 | 0.02 |
| 204 | PGM3 | 1.12 | 3.28 | 2.16 | 2.88E-05 | 0.00 |
| 205 | BLNK | 0.83 | 8.41 | 7.58 | 5.54E-03 | 0.04 |
| 206 | TBC1D9 | 0.69 | 3.16 | 2.47 | 5.38E-03 | 0.04 |
| 207 | H6PD | 8.60 | 14.52 | 5.92 | 6.07E-03 | 0.05 |
| 208 | RERE | 11.02 | 18.42 | 7.39 | 6.77E-04 | 0.01 |
| 209 | PIAS3 | 4.83 | 7.66 | 2.83 | 1.15E-03 | 0.02 |
| 210 | CKAP4 | 1.08 | 8.28 | 7.19 | 1.63E-03 | 0.02 |
| 211 | SIRPB1 | 0.90 | 12.31 | 11.41 | 1.63E-03 | 0.02 |
| 212 | EEPD1 | 1.61 | 3.76 | 2.15 | 3.22E-04 | 0.01 |
| 213 | ASMTL-AS1 | 7.77 | 12.72 | 4.95 | 4.07E-03 | 0.04 |
| 214 | SEMA4D | 8.03 | 14.86 | 6.83 | 1.20E-04 | 0.01 |
| 215 | MAFK | 4.76 | 10.03 | 5.27 | 1.11E-03 | 0.02 |
| 216 | CTNND1 | 5.23 | 12.47 | 7.25 | 5.43E-04 | 0.01 |
| 217 | KPTN | 2.22 | 4.88 | 2.66 | 1.59E-04 | 0.01 |
| 218 | PILRA | 4.12 | 18.18 | 14.06 | 1.20E-03 | 0.02 |
| 219 | PURA | 1.44 | 4.45 | 3.01 | 2.87E-04 | 0.01 |
| 220 | AC073655.2 | 1.04 | 3.78 | 2.74 | 2.27E-04 | 0.01 |
| 221 | PELI1 | 1.97 | 12.12 | 10.15 | 5.26E-05 | 0.00 |
| 222 | CDKN2D | 2.71 | 13.25 | 10.55 | 1.94E-05 | 0.00 |
| 223 | ELF4 | 19.54 | 40.16 | 20.62 | 2.19E-04 | 0.01 |
| 224 | IGSF6 | 4.06 | 13.19 | 9.12 | 7.05E-03 | 0.05 |
| 225 | TFE3 | 10.07 | 17.29 | 7.22 | 2.68E-03 | 0.03 |
| 226 | STK4 | 15.75 | 22.77 | 7.03 | 1.99E-03 | 0.02 |
| 227 | BMPR2 | 0.37 | 3.01 | 2.64 | 2.87E-04 | 0.01 |
| 228 | AL627309.7 | 2.38 | 16.35 | 13.97 | 5.71E-03 | 0.04 |
| 229 | ADAMTS10 | 0.64 | 5.58 | 4.94 | 2.35E-03 | 0.03 |
| 230 | PLP2 | 37.71 | 131.37 | 93.66 | 7.99E-05 | 0.00 |
| 231 | TNFSF8 | 2.04 | 6.36 | 4.32 | 3.36E-03 | 0.03 |
| 232 | SLC43A2 | 0.62 | 5.79 | 5.17 | 2.88E-05 | 0.00 |
| 233 | SH3BP1 | 9.61 | 20.08 | 10.47 | 3.26E-03 | 0.03 |
| 234 | WDFY4 | 4.83 | 16.58 | 11.75 | 6.22E-05 | 0.00 |
| 235 | BCL2L11 | 1.60 | 4.56 | 2.95 | 1.47E-03 | 0.02 |
| 236 | MARK3 | 13.45 | 17.35 | 3.90 | 9.35E-04 | 0.02 |
| 237 | TRGC1 | 1.79 | 11.51 | 9.72 | 2.10E-04 | 0.01 |
| 238 | SMOX | 1.14 | 4.46 | 3.32 | 2.87E-04 | 0.01 |
| 239 | SPG21 | 19.35 | 25.66 | 6.31 | 5.71E-03 | 0.04 |
| 240 | FAM198B | 1.21 | 7.43 | 6.22 | 5.89E-03 | 0.04 |
| 241 | ID2 | 4.65 | 25.19 | 20.54 | 2.98E-04 | 0.01 |
| 242 | IGHD4-17 | 1.32 | 66.74 | 65.43 | 3.12E-04 | 0.01 |
| 243 | SLC43A3 | 6.34 | 18.06 | 11.72 | 1.18E-05 | 0.00 |
| 244 | AC092384.2 | 1.10 | 3.35 | 2.25 | 1.24E-03 | 0.02 |
| 245 | ELMOD3 | 2.90 | 5.14 | 2.24 | 1.36E-04 | 0.01 |
| 246 | FYN | 3.02 | 9.82 | 6.79 | 2.98E-04 | 0.01 |
| 247 | AOAH | 3.50 | 37.83 | 34.34 | 5.49E-05 | 0.00 |
| 248 | C6orf89 | 8.13 | 12.07 | 3.93 | 5.64E-04 | 0.01 |
| 249 | HOXB-AS1 | 0.12 | 3.47 | 3.36 | 5.24E-04 | 0.01 |
| 250 | E2F2 | 2.75 | 6.07 | 3.32 | 3.47E-03 | 0.03 |
| 251 | SLC9A6 | 3.71 | 5.93 | 2.22 | 7.02E-04 | 0.01 |
| 252 | HK3 | 1.25 | 25.65 | 24.39 | 2.28E-03 | 0.03 |
| 253 | PDLIM2 | 0.94 | 3.38 | 2.44 | 7.99E-05 | 0.00 |
| 254 | CD33 | 8.46 | 22.32 | 13.86 | 1.63E-03 | 0.02 |
| 255 | PRKD3 | 1.46 | 6.78 | 5.31 | 4.86E-04 | 0.01 |
| 256 | IRF8 | 4.81 | 30.01 | 25.20 | 3.36E-03 | 0.03 |
| 257 | GPR183 | 5.12 | 29.55 | 24.43 | 4.63E-05 | 0.00 |
| 258 | ZNF414 | 5.65 | 8.91 | 3.26 | 6.85E-03 | 0.05 |
| 259 | NCOR2 | 13.35 | 21.05 | 7.70 | 3.82E-03 | 0.03 |
| 260 | CD1D | 0.95 | 11.39 | 10.44 | 2.68E-03 | 0.03 |
| 261 | NR4A2 | 3.72 | 20.00 | 16.28 | 9.02E-04 | 0.01 |
| 262 | ANO7 | 0.24 | 3.09 | 2.85 | 9.35E-04 | 0.02 |
| 263 | AC018529.2 | 2.27 | 5.47 | 3.20 | 6.77E-05 | 0.00 |
| 264 | HOXB4 | 0.08 | 7.78 | 7.70 | 2.98E-04 | 0.01 |
| 265 | NAPA | 8.20 | 12.75 | 4.55 | 1.42E-03 | 0.02 |
| 266 | AP1S2 | 5.00 | 17.01 | 12.01 | 5.04E-05 | 0.00 |
| 267 | RNU6ATAC6P | 0.07 | 26.76 | 26.69 | 2.66E-04 | 0.01 |
| 268 | ERMP1 | 9.68 | 17.80 | 8.12 | 2.20E-03 | 0.02 |
| 269 | S100A12 | 2.76 | 71.46 | 68.70 | 3.36E-03 | 0.03 |
| 270 | IGHD1-26 | 0.22 | 23.57 | 23.35 | 8.03E-04 | 0.01 |
| 271 | SNORA77 | 2.59 | 11.85 | 9.26 | 1.29E-05 | 0.00 |
| 272 | CYSTM1 | 0.98 | 3.63 | 2.65 | 6.77E-04 | 0.01 |
| 273 | SLED1 | 0.61 | 5.37 | 4.76 | 1.28E-03 | 0.02 |
| 274 | CD109 | 0.25 | 5.26 | 5.01 | 9.68E-04 | 0.02 |
| 275 | NCF2 | 9.18 | 51.22 | 42.04 | 3.47E-03 | 0.03 |
| 276 | HOXB2 | 0.19 | 7.54 | 7.35 | 6.77E-04 | 0.01 |
| 277 | NCF1C | 0.74 | 9.86 | 9.11 | 3.10E-04 | 0.01 |
| 278 | FCER1G | 8.90 | 124.00 | 115.10 | 6.49E-05 | 0.00 |
| 279 | TNRC6A | 7.52 | 10.15 | 2.63 | 2.68E-03 | 0.03 |
| 280 | ALDH2 | 0.13 | 3.02 | 2.90 | 3.90E-05 | 0.00 |
| 281 | MIR4489 | 1.19 | 3.71 | 2.53 | 7.82E-04 | 0.01 |
| 282 | AC141424.1 | 2.05 | 13.01 | 10.96 | 2.55E-04 | 0.01 |
| 283 | IL1R2 | 0.07 | 4.84 | 4.78 | 4.07E-03 | 0.04 |
| 284 | HRH2 | 3.30 | 13.41 | 10.11 | 1.15E-03 | 0.02 |
| 285 | RRAGA | 17.08 | 21.58 | 4.50 | 3.15E-03 | 0.03 |
| 286 | CEP135 | 4.20 | 6.93 | 2.73 | 1.63E-03 | 0.02 |
| 287 | IGHD4-4 | 0.58 | 95.31 | 94.73 | 5.81E-05 | 0.00 |
| 288 | KCTD9P1 | 2.33 | 4.99 | 2.66 | 5.71E-03 | 0.04 |
| 289 | PRDM16 | 0.01 | 2.51 | 2.50 | 2.51E-03 | 0.03 |
| 290 | SELL | 58.98 | 171.40 | 112.42 | 6.65E-03 | 0.05 |
| 291 | PCCA | 0.73 | 3.37 | 2.64 | 2.27E-04 | 0.01 |
| 292 | ARHGAP27P1-BPTFP1-KPNA2P3 | 3.19 | 5.78 | 2.59 | 4.86E-04 | 0.01 |
| 293 | TNFSF10 | 2.79 | 15.30 | 12.50 | 2.27E-04 | 0.01 |
| 294 | DDIT4 | 3.08 | 18.32 | 15.24 | 7.05E-03 | 0.05 |
| 295 | CD14 | 1.92 | 43.06 | 41.13 | 7.05E-03 | 0.05 |
| 296 | ITGB2 | 18.63 | 105.44 | 86.81 | 4.83E-05 | 0.00 |
| 297 | ERBIN | 9.98 | 16.74 | 6.76 | 1.04E-03 | 0.02 |
| 298 | RIN2 | 0.23 | 2.68 | 2.45 | 1.30E-04 | 0.01 |
| 299 | SUSD6 | 10.73 | 17.58 | 6.85 | 1.59E-04 | 0.01 |
| 300 | U51244.1 | 0.77 | 3.81 | 3.04 | 5.43E-04 | 0.01 |
| 301 | SLC25A28 | 18.02 | 29.20 | 11.17 | 5.64E-04 | 0.01 |
| 302 | IQCE | 0.64 | 2.70 | 2.06 | 1.13E-05 | 0.00 |
| 303 | RN7SKP26 | 2.02 | 4.02 | 2.00 | 5.21E-03 | 0.04 |
| 304 | GABARAPL1 | 0.74 | 6.98 | 6.24 | 9.83E-06 | 0.00 |
| 305 | AC127502.2 | 3.55 | 8.06 | 4.51 | 3.36E-03 | 0.03 |
| 306 | TCTEX1D1 | 0.80 | 8.04 | 7.24 | 3.36E-03 | 0.03 |
| 307 | GPD2 | 4.65 | 11.36 | 6.71 | 6.77E-05 | 0.00 |
| 308 | TRABD | 15.33 | 35.88 | 20.55 | 2.65E-04 | 0.01 |
| 309 | RAP1GAP2 | 6.27 | 16.69 | 10.41 | 2.27E-04 | 0.01 |
| 310 | AC132812.1 | 9.99 | 19.34 | 9.35 | 1.15E-03 | 0.02 |
| 311 | SH3TC1 | 2.24 | 7.06 | 4.82 | 5.21E-03 | 0.04 |
| 312 | AC069549.1 | 1.21 | 9.43 | 8.22 | 1.66E-04 | 0.01 |
| 313 | ABI3 | 0.87 | 6.24 | 5.37 | 5.43E-04 | 0.01 |
| 314 | NIPA1 | 0.47 | 4.90 | 4.43 | 1.35E-05 | 0.00 |
| 315 | DOCK1 | 0.03 | 4.87 | 4.84 | 1.47E-04 | 0.01 |
| 316 | LPIN1 | 5.04 | 10.13 | 5.09 | 4.61E-03 | 0.04 |
| 317 | AL157938.1 | 0.87 | 3.06 | 2.19 | 3.60E-04 | 0.01 |
| 318 | RGS14 | 6.13 | 16.85 | 10.72 | 4.19E-04 | 0.01 |
| 319 | HSPA7 | 0.19 | 5.81 | 5.62 | 3.22E-04 | 0.01 |
| 320 | ARHGAP18 | 3.01 | 5.95 | 2.94 | 2.13E-03 | 0.02 |
| 321 | FGD3 | 12.64 | 22.61 | 9.98 | 1.63E-03 | 0.02 |
| 322 | HPCAL1 | 7.31 | 11.69 | 4.38 | 5.54E-03 | 0.04 |
| 323 | VNN2 | 1.68 | 12.93 | 11.25 | 4.75E-03 | 0.04 |
| 324 | NAPSB | 0.85 | 13.87 | 13.02 | 6.53E-04 | 0.01 |
| 325 | BRAT1 | 5.96 | 10.98 | 5.02 | 2.68E-03 | 0.03 |
| 326 | ZC3H12C | 0.73 | 4.16 | 3.43 | 1.08E-03 | 0.02 |
| 327 | NBR1 | 6.78 | 12.29 | 5.50 | 1.30E-04 | 0.01 |
| 328 | PFKFB3 | 4.82 | 11.01 | 6.19 | 3.89E-04 | 0.01 |
| 329 | MIR1-1HG-AS1 | 0.02 | 2.77 | 2.75 | 1.62E-03 | 0.02 |
| 330 | TBC1D2 | 5.14 | 11.61 | 6.47 | 5.54E-03 | 0.04 |
| 331 | ARHGEF1 | 25.84 | 44.03 | 18.19 | 4.07E-03 | 0.04 |
| 332 | IGHD2-8 | 0.00 | 32.40 | 32.40 | 5.59E-05 | 0.00 |
| 333 | OSBPL3 | 3.28 | 9.85 | 6.58 | 1.94E-04 | 0.01 |
| 334 | NHSL2 | 0.49 | 6.83 | 6.34 | 6.49E-05 | 0.00 |
| 335 | PRKACA | 5.99 | 10.50 | 4.50 | 1.74E-03 | 0.02 |
| 336 | PPP1R16B | 1.70 | 6.35 | 4.64 | 6.07E-03 | 0.05 |
| 337 | LRP1 | 0.98 | 15.88 | 14.91 | 2.35E-03 | 0.03 |
| 338 | SLC1A4 | 5.21 | 11.48 | 6.27 | 3.34E-04 | 0.01 |
| 339 | MIR4518 | 2.43 | 7.42 | 4.99 | 3.15E-03 | 0.03 |
| 340 | DECR1 | 10.04 | 14.44 | 4.40 | 2.77E-03 | 0.03 |
| 341 | AC011120.1 | 3.97 | 8.34 | 4.37 | 2.35E-03 | 0.03 |
| 342 | ANKRD44-IT1 | 5.86 | 12.17 | 6.31 | 2.60E-03 | 0.03 |
| 343 | TNFRSF1B | 7.87 | 50.27 | 42.39 | 3.47E-04 | 0.01 |
| 344 | MGAT3 | 0.26 | 2.29 | 2.03 | 2.98E-04 | 0.01 |
| 345 | GPR108 | 11.67 | 16.70 | 5.03 | 7.05E-03 | 0.05 |
| 346 | HCLS1 | 30.84 | 51.65 | 20.81 | 2.10E-04 | 0.01 |
| 347 | AC131097.4 | 1.06 | 3.24 | 2.18 | 6.65E-03 | 0.05 |
| 348 | SIGLEC10 | 2.02 | 9.66 | 7.63 | 2.46E-04 | 0.01 |
| 349 | RNASE2 | 65.11 | 382.42 | 317.31 | 5.54E-03 | 0.04 |
| 350 | VCL | 15.89 | 25.20 | 9.30 | 3.47E-03 | 0.03 |
| 351 | RNU6-226P | 0.93 | 4.19 | 3.26 | 5.21E-03 | 0.04 |
| 352 | IQSEC2 | 0.60 | 2.99 | 2.39 | 5.43E-04 | 0.01 |
| 353 | PA2G4P6 | 2.96 | 5.79 | 2.83 | 2.13E-03 | 0.02 |
| 354 | CX3CR1 | 3.15 | 33.64 | 30.49 | 1.33E-03 | 0.02 |
| 355 | CSF2RA | 2.20 | 17.30 | 15.10 | 3.90E-05 | 0.00 |
| 356 | AC131009.4 | 1.84 | 4.30 | 2.47 | 9.82E-05 | 0.01 |
| 357 | AC245884.12 | 0.15 | 7.94 | 7.79 | 3.41E-03 | 0.03 |
| 358 | NINJ2 | 1.07 | 4.65 | 3.59 | 4.25E-05 | 0.00 |
| 359 | TGFBR1 | 6.92 | 10.93 | 4.01 | 4.07E-03 | 0.04 |
| 360 | GRAMD4 | 10.05 | 18.47 | 8.42 | 3.82E-03 | 0.03 |
| 361 | CAMK1 | 0.11 | 2.28 | 2.16 | 1.08E-05 | 0.00 |
| 362 | FPR1 | 0.48 | 15.24 | 14.76 | 7.28E-04 | 0.01 |
| 363 | SMCO4 | 2.36 | 8.23 | 5.87 | 1.93E-03 | 0.02 |
| 364 | IGHJ2P | 0.34 | 16.90 | 16.56 | 1.53E-03 | 0.02 |
| 365 | ELF2 | 10.19 | 14.78 | 4.59 | 6.29E-04 | 0.01 |
| 366 | C1orf162 | 3.39 | 31.86 | 28.47 | 2.12E-05 | 0.00 |
| 367 | STAB1 | 2.67 | 54.01 | 51.34 | 2.46E-04 | 0.01 |
| 368 | TMEM273 | 0.38 | 4.43 | 4.05 | 1.74E-03 | 0.02 |
| 369 | GP1BA | 2.37 | 6.37 | 4.00 | 2.46E-04 | 0.01 |
| 370 | IGF2R | 1.51 | 8.87 | 7.37 | 9.02E-04 | 0.01 |
| 371 | SP100 | 9.71 | 13.81 | 4.10 | 1.68E-03 | 0.02 |
| 372 | ZFAND2B | 5.86 | 9.30 | 3.45 | 4.75E-03 | 0.04 |
| 373 | GLIPR2 | 2.81 | 25.61 | 22.80 | 1.03E-05 | 0.00 |
| 374 | HTT | 9.47 | 13.44 | 3.98 | 4.61E-03 | 0.04 |
| 375 | SYNJ1 | 3.66 | 5.99 | 2.32 | 6.53E-04 | 0.01 |
| 376 | SERTAD2 | 16.12 | 23.46 | 7.34 | 3.47E-03 | 0.03 |
| 377 | RGS19 | 21.07 | 39.41 | 18.33 | 4.20E-03 | 0.04 |
| 378 | NECTIN1 | 0.85 | 7.19 | 6.34 | 4.83E-05 | 0.00 |
| 379 | IGHD6-13 | 0.54 | 68.03 | 67.49 | 7.30E-05 | 0.00 |
| 380 | MAP7 | 2.09 | 9.80 | 7.70 | 1.68E-03 | 0.02 |
| 381 | LAPTM5 | 72.66 | 355.13 | 282.47 | 9.83E-06 | 0.00 |
| 382 | RXFP1 | 0.17 | 4.09 | 3.91 | 4.90E-03 | 0.04 |
| 383 | ZNF664 | 6.85 | 11.98 | 5.13 | 1.20E-03 | 0.02 |
| 384 | CTSD | 33.90 | 138.66 | 104.76 | 8.33E-05 | 0.00 |
| 385 | HIVEP3 | 1.54 | 4.55 | 3.01 | 3.60E-04 | 0.01 |
| 386 | VDR | 0.97 | 4.08 | 3.11 | 2.35E-03 | 0.03 |
| 387 | EFHC2 | 0.18 | 2.26 | 2.08 | 3.60E-04 | 0.01 |
| 388 | TRAF4 | 2.16 | 5.57 | 3.41 | 8.70E-04 | 0.01 |
| 389 | AC145207.8 | 1.93 | 5.01 | 3.08 | 3.01E-05 | 0.00 |
| 390 | CLEC4A | 0.84 | 8.84 | 8.00 | 2.65E-04 | 0.01 |
| 391 | SLC40A1 | 3.49 | 35.45 | 31.96 | 2.19E-04 | 0.01 |
| 392 | MCEMP1 | 1.66 | 10.21 | 8.55 | 5.06E-03 | 0.04 |
| 393 | IL3RA | 19.62 | 43.22 | 23.59 | 8.40E-04 | 0.01 |
| 394 | AC246787.2 | 0.23 | 27.74 | 27.51 | 3.01E-05 | 0.00 |
| 395 | SLC3A2 | 12.14 | 16.92 | 4.79 | 4.33E-03 | 0.04 |
| 396 | FPR2 | 0.23 | 3.48 | 3.25 | 3.70E-03 | 0.03 |
| 397 | STX11 | 4.10 | 20.62 | 16.51 | 1.20E-03 | 0.02 |
| 398 | LEMD2 | 6.01 | 8.03 | 2.01 | 1.47E-03 | 0.02 |
| 399 | SLA | 3.04 | 15.63 | 12.58 | 2.76E-05 | 0.00 |
| 400 | TACC1 | 10.93 | 17.41 | 6.48 | 4.86E-04 | 0.01 |
| 401 | TSPAN2 | 0.64 | 5.81 | 5.17 | 3.60E-04 | 0.01 |
| 402 | ARHGEF11 | 0.35 | 6.58 | 6.23 | 1.73E-04 | 0.01 |
| 403 | MAP1A | 1.57 | 8.07 | 6.50 | 3.94E-03 | 0.04 |
| 404 | CMTM4 | 0.68 | 4.28 | 3.59 | 6.49E-05 | 0.00 |
| 405 | TYROBP | 49.17 | 183.53 | 134.36 | 3.47E-03 | 0.03 |
| 406 | S100A6 | 16.80 | 92.10 | 75.30 | 2.02E-04 | 0.01 |
| 407 | ZNF652 | 6.58 | 10.48 | 3.90 | 9.68E-04 | 0.02 |
| 408 | ARHGEF10L | 0.10 | 2.80 | 2.70 | 2.35E-03 | 0.03 |
| 409 | PLXDC2 | 0.96 | 8.29 | 7.33 | 8.33E-05 | 0.00 |
| 410 | MICAL2 | 1.22 | 3.64 | 2.42 | 5.06E-03 | 0.04 |
| 411 | SNX9 | 0.67 | 4.11 | 3.44 | 2.87E-04 | 0.01 |
| 412 | SENCR | 2.24 | 4.46 | 2.22 | 7.82E-04 | 0.01 |
| 413 | CD86 | 0.78 | 11.30 | 10.52 | 1.59E-04 | 0.01 |
| 414 | CTSG | 7.79 | 165.21 | 157.43 | 3.15E-03 | 0.03 |
| 415 | PLEKHO2 | 8.11 | 17.03 | 8.92 | 5.43E-04 | 0.01 |
| 416 | SEL1L3 | 2.43 | 13.62 | 11.20 | 2.27E-04 | 0.01 |
| 417 | IGHD4-23 | 0.70 | 22.06 | 21.36 | 3.21E-03 | 0.03 |
| 418 | SERINC1 | 29.85 | 43.96 | 14.11 | 1.52E-03 | 0.02 |
| 419 | PBX3 | 4.49 | 20.28 | 15.79 | 4.47E-03 | 0.04 |
| 420 | ADGRE2 | 7.70 | 23.46 | 15.75 | 1.36E-04 | 0.01 |
| 421 | GAB3 | 12.71 | 21.84 | 9.13 | 1.86E-03 | 0.02 |
| 422 | TMEM65 | 3.14 | 8.32 | 5.19 | 1.52E-03 | 0.02 |
| 423 | RAB3D | 10.16 | 23.56 | 13.40 | 7.02E-04 | 0.01 |
| 424 | AC100810.1 | 3.36 | 5.44 | 2.07 | 3.70E-03 | 0.03 |
| 425 | METRNL | 1.08 | 7.44 | 6.36 | 1.02E-04 | 0.01 |
| 426 | TMEM229B | 0.80 | 3.25 | 2.45 | 8.68E-05 | 0.00 |
| 427 | IGHD2-15 | 0.78 | 39.15 | 38.37 | 9.97E-05 | 0.01 |
| 428 | OGG1 | 0.85 | 4.76 | 3.91 | 8.96E-06 | 0.00 |
| 429 | MMP9 | 0.11 | 2.65 | 2.54 | 4.33E-03 | 0.04 |
| 430 | SNX10 | 2.78 | 16.39 | 13.62 | 5.72E-05 | 0.00 |
| 431 | MIR7848 | 1.94 | 10.57 | 8.62 | 5.72E-05 | 0.00 |
| 432 | SCPEP1 | 5.85 | 27.87 | 22.02 | 5.21E-03 | 0.04 |
| 433 | OBSL1 | 0.67 | 4.86 | 4.19 | 4.47E-03 | 0.04 |
| 434 | RHBDF1 | 0.11 | 2.35 | 2.24 | 4.44E-05 | 0.00 |
| 435 | SCUBE1 | 0.31 | 3.58 | 3.26 | 2.43E-03 | 0.03 |
| 436 | PRKCE | 0.73 | 3.09 | 2.36 | 1.30E-04 | 0.01 |
| 437 | JDP2 | 1.85 | 4.88 | 3.03 | 1.08E-03 | 0.02 |
| 438 | ACP5 | 0.90 | 3.24 | 2.34 | 6.07E-04 | 0.01 |
| 439 | RAB8A | 15.52 | 20.43 | 4.91 | 1.20E-03 | 0.02 |
| 440 | SIGLEC22P | 3.31 | 10.40 | 7.09 | 7.05E-03 | 0.05 |
| 441 | MIR548J | 1.43 | 3.99 | 2.56 | 1.08E-03 | 0.02 |
| 442 | LINC01475 | 0.00 | 5.01 | 5.01 | 1.24E-03 | 0.02 |
| 443 | C11orf21 | 2.37 | 30.65 | 28.28 | 1.18E-05 | 0.00 |
| 444 | TRAFD1 | 7.73 | 11.00 | 3.26 | 1.68E-03 | 0.02 |
| 445 | SIPA1 | 17.80 | 43.18 | 25.38 | 7.99E-05 | 0.00 |
| 446 | LCAT | 3.23 | 5.93 | 2.70 | 1.47E-04 | 0.01 |
| 447 | PECAM1 | 21.33 | 47.19 | 25.86 | 2.20E-03 | 0.02 |
| 448 | AL391121.1 | 0.93 | 3.70 | 2.77 | 3.74E-05 | 0.00 |
| 449 | PLEKHM3 | 2.56 | 4.73 | 2.16 | 2.36E-04 | 0.01 |
| 450 | AP001266.2 | 1.13 | 4.07 | 2.94 | 4.61E-03 | 0.04 |
| 451 | COBLL1 | 0.23 | 2.36 | 2.13 | 8.40E-04 | 0.01 |
| 452 | IGHD5-24 | 0.00 | 27.36 | 27.36 | 2.50E-04 | 0.01 |
| 453 | PPIAP53 | 1.71 | 6.85 | 5.14 | 2.36E-04 | 0.01 |
| 454 | AL513343.1 | 1.51 | 4.07 | 2.56 | 1.52E-03 | 0.02 |
| 455 | SRSF5 | 57.84 | 82.07 | 24.23 | 3.47E-03 | 0.03 |
| 456 | LMBRD1 | 11.10 | 15.64 | 4.54 | 5.38E-03 | 0.04 |
| 457 | IDNK | 2.26 | 4.87 | 2.61 | 9.43E-05 | 0.00 |
| 458 | CRAT | 4.02 | 8.54 | 4.51 | 1.74E-03 | 0.02 |
| 459 | IGHD3-16 | 1.17 | 113.14 | 111.97 | 4.07E-05 | 0.00 |
| 460 | RBMS1 | 6.27 | 9.74 | 3.48 | 5.38E-03 | 0.04 |
| 461 | IGHD2-2 | 0.80 | 42.62 | 41.82 | 4.07E-05 | 0.00 |
| 462 | ABCA7 | 2.27 | 16.56 | 14.28 | 1.18E-05 | 0.00 |
| 463 | AL133415.1 | 1.60 | 8.34 | 6.73 | 1.16E-04 | 0.01 |
| 464 | CCDC69 | 11.28 | 21.99 | 10.71 | 2.96E-03 | 0.03 |
| 465 | IL10RB | 10.97 | 22.39 | 11.42 | 2.27E-04 | 0.01 |
| 466 | LGALS1 | 28.28 | 137.31 | 109.03 | 8.10E-04 | 0.01 |
| 467 | WDR81 | 7.13 | 12.22 | 5.09 | 4.33E-03 | 0.04 |
| 468 | GRK6 | 13.88 | 26.82 | 12.95 | 2.65E-04 | 0.01 |
| 469 | CXCR4 | 39.35 | 141.40 | 102.04 | 5.43E-04 | 0.01 |
| 470 | HEBP1 | 4.60 | 8.28 | 3.68 | 3.74E-04 | 0.01 |
| 471 | ABCA1 | 0.33 | 3.52 | 3.19 | 1.69E-05 | 0.00 |
| 472 | NFAM1 | 2.23 | 17.79 | 15.56 | 3.15E-03 | 0.03 |
| 473 | P2RY14 | 0.89 | 4.00 | 3.11 | 4.61E-03 | 0.04 |
| 474 | ST6GALNAC4 | 2.72 | 7.92 | 5.20 | 2.98E-04 | 0.01 |
| 475 | CDA | 0.98 | 18.82 | 17.84 | 1.24E-03 | 0.02 |
| 476 | CCL1 | 0.00 | 2.26 | 2.26 | 1.82E-03 | 0.02 |
| 477 | TFEB | 1.67 | 4.50 | 2.82 | 6.45E-03 | 0.05 |
| 478 | ZCCHC2 | 2.96 | 5.42 | 2.47 | 4.52E-04 | 0.01 |
| 479 | RASSF2 | 13.76 | 43.30 | 29.54 | 2.64E-05 | 0.00 |
| 480 | AC004656.1 | 3.99 | 6.54 | 2.55 | 2.20E-03 | 0.02 |
| 481 | CD164 | 43.08 | 87.50 | 44.42 | 1.24E-03 | 0.02 |
| 482 | TCEAL9 | 2.33 | 8.96 | 6.64 | 2.77E-03 | 0.03 |
| 483 | MTMR1 | 3.61 | 7.85 | 4.24 | 4.25E-05 | 0.00 |
| 484 | DENND6B | 2.40 | 5.31 | 2.91 | 1.74E-03 | 0.02 |
| 485 | RASSF5 | 4.80 | 15.87 | 11.07 | 9.39E-06 | 0.00 |
| 486 | RAB24 | 2.33 | 7.45 | 5.12 | 7.06E-05 | 0.00 |
| 487 | SKIL | 0.78 | 2.86 | 2.07 | 1.80E-04 | 0.01 |
| 488 | ARHGEF7 | 4.59 | 8.08 | 3.48 | 7.36E-05 | 0.00 |
| 489 | H2AFJ | 0.51 | 4.28 | 3.77 | 1.02E-04 | 0.01 |
| 490 | INPP4A | 3.89 | 8.78 | 4.89 | 5.49E-05 | 0.00 |
| 491 | DTX4 | 0.59 | 3.23 | 2.65 | 2.06E-03 | 0.02 |
| 492 | HTRA3 | 0.62 | 7.79 | 7.17 | 1.15E-03 | 0.02 |
| 493 | FAM102B | 4.09 | 7.31 | 3.22 | 1.42E-03 | 0.02 |
| 494 | TRIM8 | 8.98 | 31.31 | 22.33 | 3.01E-05 | 0.00 |
| 495 | TSPAN17 | 2.78 | 5.47 | 2.69 | 1.33E-03 | 0.02 |
| 496 | NINJ1 | 1.50 | 12.27 | 10.77 | 3.74E-05 | 0.00 |
| 497 | GPR18 | 3.52 | 6.37 | 2.85 | 6.85E-03 | 0.05 |
| 498 | C3orf80 | 0.04 | 15.27 | 15.23 | 1.41E-05 | 0.00 |
| 499 | SLC22A20P | 0.23 | 3.19 | 2.97 | 2.76E-05 | 0.00 |
| 500 | ETS2 | 15.25 | 41.08 | 25.83 | 4.63E-05 | 0.00 |
| 501 | FOSL1 | 2.79 | 9.78 | 6.99 | 1.86E-03 | 0.02 |
| 502 | ZYG11B | 3.94 | 6.90 | 2.96 | 2.10E-04 | 0.01 |
| 503 | TTC38 | 6.87 | 11.99 | 5.11 | 3.58E-03 | 0.03 |
| 504 | MXD1 | 9.18 | 25.65 | 16.48 | 1.20E-04 | 0.01 |
| 505 | PQLC2 | 4.06 | 9.33 | 5.27 | 2.55E-04 | 0.01 |
| 506 | RIPK3 | 3.30 | 6.77 | 3.47 | 1.00E-03 | 0.02 |
| 507 | PRKCD | 9.23 | 53.85 | 44.63 | 2.88E-05 | 0.00 |
| 508 | IGHD2-21 | 0.44 | 26.55 | 26.11 | 2.16E-04 | 0.01 |
| 509 | USB1 | 6.08 | 8.55 | 2.47 | 5.38E-03 | 0.04 |
| 510 | AC073655.1 | 0.77 | 4.50 | 3.72 | 1.20E-04 | 0.01 |
| 511 | CARD19 | 5.61 | 22.46 | 16.85 | 6.49E-05 | 0.00 |
| 512 | AC104232.1 | 0.08 | 11.81 | 11.72 | 3.08E-04 | 0.01 |
| 513 | AC016292.2 | 0.23 | 2.67 | 2.44 | 5.15E-05 | 0.00 |
| 514 | MBD2 | 8.80 | 12.95 | 4.15 | 2.35E-03 | 0.03 |
| 515 | SLC7A7 | 1.02 | 12.30 | 11.29 | 1.53E-04 | 0.01 |
| 516 | AL022328.3 | 3.11 | 8.21 | 5.10 | 5.26E-05 | 0.00 |
| 517 | AF064858.3 | 0.50 | 2.85 | 2.35 | 1.66E-04 | 0.01 |
| 518 | ICAM2 | 6.03 | 9.58 | 3.55 | 4.90E-03 | 0.04 |
| 519 | CYTH4 | 3.82 | 25.75 | 21.93 | 2.31E-05 | 0.00 |
| 520 | CPEB2 | 0.99 | 4.12 | 3.13 | 9.43E-05 | 0.00 |
| 521 | IGHD6-19 | 1.50 | 77.45 | 75.95 | 2.54E-04 | 0.01 |
| 522 | ATP6V1A | 14.43 | 25.69 | 11.26 | 3.10E-04 | 0.01 |
| 523 | SMPD2 | 4.07 | 6.13 | 2.06 | 1.99E-03 | 0.02 |
| 524 | ZNF318 | 3.55 | 8.00 | 4.45 | 4.69E-04 | 0.01 |
| 525 | AL023284.4 | 0.17 | 3.25 | 3.09 | 1.02E-04 | 0.01 |
| 526 | EVI2B | 59.36 | 129.98 | 70.62 | 3.15E-03 | 0.03 |
| 527 | PI4K2A | 0.58 | 2.80 | 2.22 | 4.04E-04 | 0.01 |
| 528 | SNAP29 | 8.09 | 12.36 | 4.27 | 1.47E-04 | 0.01 |
| 529 | SGSH | 0.70 | 4.21 | 3.51 | 1.93E-03 | 0.02 |
| 530 | HOXA10-AS | 0.29 | 4.56 | 4.27 | 3.70E-04 | 0.01 |
| 531 | CLIC2 | 0.68 | 4.40 | 3.71 | 4.90E-03 | 0.04 |
| 532 | ASGR2 | 0.06 | 3.51 | 3.45 | 7.36E-05 | 0.00 |
| 533 | S100A9 | 66.67 | 999.95 | 933.27 | 6.07E-03 | 0.05 |
| 534 | ARRDC5 | 5.16 | 10.25 | 5.10 | 6.45E-03 | 0.05 |
| 535 | EHBP1L1 | 14.93 | 31.00 | 16.06 | 3.89E-04 | 0.01 |
| 536 | RLF | 7.83 | 10.78 | 2.95 | 7.05E-03 | 0.05 |
| 537 | AC005838.2 | 0.94 | 3.89 | 2.95 | 7.82E-04 | 0.01 |
| 538 | PTPN12 | 6.02 | 18.14 | 12.12 | 2.88E-05 | 0.00 |
| 539 | SH2B3 | 16.97 | 32.20 | 15.23 | 3.74E-04 | 0.01 |
| 540 | TUBB1 | 0.79 | 3.53 | 2.74 | 2.86E-03 | 0.03 |
| 541 | SLC30A1 | 3.72 | 6.44 | 2.72 | 5.21E-03 | 0.04 |
| 542 | SDSL | 1.13 | 3.90 | 2.77 | 9.35E-04 | 0.02 |
| 543 | RXRA | 1.31 | 8.08 | 6.77 | 4.83E-05 | 0.00 |
| 544 | TOP1MT | 4.48 | 10.93 | 6.45 | 1.33E-03 | 0.02 |
| 545 | IGHD4-11 | 1.02 | 88.65 | 87.63 | 7.63E-05 | 0.00 |
| 546 | AL391863.2 | 0.35 | 2.44 | 2.09 | 1.00E-03 | 0.02 |
| 547 | AOAH-IT1 | 0.25 | 3.17 | 2.92 | 6.63E-05 | 0.00 |
| 548 | FADS1 | 3.52 | 7.85 | 4.32 | 9.35E-04 | 0.02 |
| 549 | ZCCHC24 | 0.36 | 2.43 | 2.07 | 9.43E-05 | 0.00 |
| 550 | IGHD1-20 | 0.00 | 55.89 | 55.89 | 4.18E-05 | 0.00 |
| 551 | GLTP | 6.64 | 10.38 | 3.73 | 1.24E-03 | 0.02 |
| 552 | BIN2 | 31.65 | 61.83 | 30.17 | 7.02E-04 | 0.01 |
| 553 | DYTN | 0.46 | 2.73 | 2.27 | 5.21E-03 | 0.04 |
| 554 | SC5D | 2.91 | 4.94 | 2.02 | 1.37E-03 | 0.02 |
| 555 | GDI2P2 | 0.67 | 6.33 | 5.66 | 4.07E-03 | 0.04 |
| 556 | CAPN7 | 6.47 | 9.14 | 2.66 | 4.20E-03 | 0.04 |
| 557 | AL590560.2 | 1.75 | 3.97 | 2.22 | 6.07E-03 | 0.05 |
| 558 | AC024267.3 | 3.97 | 7.81 | 3.84 | 9.68E-04 | 0.02 |
| 559 | PCYT1A | 8.06 | 10.71 | 2.65 | 9.68E-04 | 0.02 |
| 560 | PPM1M | 3.23 | 11.58 | 8.35 | 2.12E-05 | 0.00 |
| 561 | NFIL3 | 2.65 | 22.22 | 19.57 | 1.35E-05 | 0.00 |
| 562 | MIR3690 | 1.48 | 9.33 | 7.85 | 2.10E-04 | 0.01 |
| 563 | KLF13 | 11.91 | 24.43 | 12.53 | 7.36E-05 | 0.00 |
| 564 | HOXB-AS2 | 0.03 | 12.87 | 12.84 | 4.72E-05 | 0.00 |
| 565 | AC067817.2 | 1.87 | 4.10 | 2.23 | 2.28E-03 | 0.03 |
| 566 | TPST2 | 5.25 | 13.61 | 8.36 | 1.69E-05 | 0.00 |
| 567 | LTB4R2 | 3.61 | 7.03 | 3.42 | 2.20E-03 | 0.02 |
| 568 | MEF2D | 25.17 | 44.26 | 19.09 | 1.42E-03 | 0.02 |
| 569 | TOMM40L | 4.89 | 6.93 | 2.05 | 6.65E-03 | 0.05 |
| 570 | MICAL1 | 11.25 | 32.01 | 20.77 | 8.40E-04 | 0.01 |
| 571 | YY1AP1 | 20.27 | 23.91 | 3.63 | 3.05E-03 | 0.03 |
| 572 | AC010186.3 | 1.76 | 8.53 | 6.76 | 3.58E-05 | 0.00 |
| 573 | RNU6-1024P | 2.00 | 5.83 | 3.83 | 5.21E-03 | 0.04 |
| 574 | ST8SIA6-AS1 | 0.05 | 3.38 | 3.34 | 2.31E-05 | 0.00 |
| 575 | ARHGAP5 | 0.65 | 4.93 | 4.28 | 3.58E-03 | 0.03 |
| 576 | S100A8 | 25.83 | 336.51 | 310.68 | 6.85E-03 | 0.05 |
| 577 | SASH3 | 22.13 | 63.22 | 41.08 | 6.22E-05 | 0.00 |
| 578 | NBEAL2 | 25.25 | 57.04 | 31.79 | 1.93E-03 | 0.02 |
| 579 | PTPN1 | 15.32 | 22.73 | 7.41 | 1.20E-04 | 0.01 |
| 580 | MED12L | 0.33 | 3.01 | 2.68 | 1.86E-03 | 0.02 |
| 581 | PRPF38AP2 | 0.15 | 3.45 | 3.30 | 4.19E-04 | 0.01 |
| 582 | BIK | 0.83 | 5.49 | 4.66 | 6.29E-04 | 0.01 |
| 583 | HLX | 2.18 | 6.26 | 4.08 | 1.33E-03 | 0.02 |
| 584 | STIM2 | 2.89 | 5.81 | 2.91 | 2.53E-05 | 0.00 |
| 585 | RUBCN | 6.64 | 8.83 | 2.20 | 5.38E-03 | 0.04 |
| 586 | AC027104.1 | 0.53 | 3.56 | 3.03 | 2.28E-03 | 0.03 |
| 587 | SELENOP | 0.51 | 6.14 | 5.63 | 3.58E-03 | 0.03 |
| 588 | CDKN1B | 9.00 | 14.43 | 5.43 | 3.05E-03 | 0.03 |
| 589 | AC116366.2 | 1.66 | 4.40 | 2.75 | 3.36E-03 | 0.03 |
| 590 | NLRP3 | 4.78 | 15.60 | 10.83 | 3.60E-04 | 0.01 |
| 591 | RASSF7 | 1.95 | 5.40 | 3.45 | 1.53E-04 | 0.01 |
| 592 | SH3GLB2 | 2.90 | 8.35 | 5.45 | 1.94E-05 | 0.00 |
| 593 | AP001363.1 | 4.66 | 17.05 | 12.39 | 1.24E-03 | 0.02 |
| 594 | SMPD1 | 4.03 | 7.41 | 3.38 | 7.02E-04 | 0.01 |
| 595 | RAPGEF1 | 13.17 | 24.71 | 11.54 | 2.76E-04 | 0.01 |
| 596 | LGALS3 | 1.80 | 13.23 | 11.43 | 1.57E-03 | 0.02 |
| 597 | B4GALT1 | 14.94 | 23.03 | 8.09 | 2.28E-03 | 0.03 |
| 598 | LTBP1 | 0.36 | 8.36 | 8.00 | 1.00E-03 | 0.02 |
| 599 | LMO4 | 1.94 | 7.80 | 5.86 | 1.37E-03 | 0.02 |
| 600 | RIT1 | 6.92 | 12.40 | 5.47 | 8.33E-05 | 0.00 |
| 601 | SBF2 | 2.01 | 4.03 | 2.02 | 6.65E-03 | 0.05 |
| 602 | AC132872.3 | 3.13 | 6.01 | 2.88 | 9.68E-04 | 0.02 |
| 603 | PDP1 | 3.38 | 5.39 | 2.00 | 3.36E-03 | 0.03 |
| 604 | DEDD2 | 6.42 | 13.18 | 6.76 | 3.89E-04 | 0.01 |
| 605 | JMJD6 | 3.20 | 5.52 | 2.32 | 2.55E-04 | 0.01 |
| 606 | ASRGL1 | 1.07 | 4.12 | 3.05 | 3.58E-03 | 0.03 |
| 607 | C9orf72 | 3.26 | 12.52 | 9.26 | 6.77E-05 | 0.00 |
| 608 | BTBD11 | 0.73 | 4.01 | 3.28 | 1.93E-03 | 0.02 |
| 609 | AP002807.1 | 3.23 | 6.58 | 3.35 | 2.28E-03 | 0.03 |
| 610 | CD300LB | 0.56 | 7.97 | 7.41 | 2.76E-04 | 0.01 |
| 611 | TLE4 | 9.05 | 14.23 | 5.18 | 3.58E-03 | 0.03 |
| 612 | HTR1F | 0.02 | 4.72 | 4.70 | 1.23E-04 | 0.01 |
| 613 | RNU6-878P | 1.77 | 30.08 | 28.31 | 1.08E-05 | 0.00 |
| 614 | PSTPIP1 | 2.26 | 12.03 | 9.78 | 2.76E-05 | 0.00 |
| 615 | TXNDC11 | 12.39 | 16.26 | 3.87 | 6.07E-03 | 0.05 |
| 616 | PLEC | 9.02 | 28.29 | 19.27 | 2.60E-03 | 0.03 |
| 617 | AC068790.4 | 1.01 | 3.43 | 2.42 | 1.74E-03 | 0.02 |
| 618 | GPR132 | 1.64 | 6.68 | 5.05 | 1.47E-04 | 0.01 |
| 619 | FUT7 | 0.29 | 4.81 | 4.52 | 1.13E-05 | 0.00 |
| 620 | TNNI2 | 0.13 | 3.46 | 3.33 | 5.49E-05 | 0.00 |
| 621 | FNBP1 | 29.12 | 54.14 | 25.02 | 4.44E-05 | 0.00 |
| 622 | LRRK2 | 0.37 | 4.25 | 3.87 | 3.82E-03 | 0.03 |
| 623 | ATP13A2 | 3.71 | 12.58 | 8.87 | 7.06E-05 | 0.00 |
| 624 | MIR4420 | 1.45 | 19.34 | 17.89 | 1.41E-05 | 0.00 |
| 625 | LPAR4 | 0.57 | 3.17 | 2.61 | 6.65E-03 | 0.05 |
| 626 | LILRA1 | 2.36 | 13.37 | 11.02 | 1.93E-03 | 0.02 |
| 627 | AC004865.2 | 2.10 | 7.35 | 5.24 | 9.82E-05 | 0.01 |
| 628 | CPVL | 2.81 | 16.68 | 13.87 | 7.28E-04 | 0.01 |
| 629 | AC148476.1 | 0.05 | 2.09 | 2.04 | 2.31E-05 | 0.00 |
| 630 | CXCL16 | 0.44 | 5.25 | 4.81 | 2.46E-04 | 0.01 |
| 631 | VWF | 0.19 | 3.33 | 3.14 | 7.67E-05 | 0.00 |
| 632 | TNFAIP2 | 3.00 | 41.60 | 38.60 | 1.85E-05 | 0.00 |
| 633 | RNF141 | 3.78 | 6.82 | 3.03 | 4.19E-04 | 0.01 |
| 634 | MIR548AT | 0.48 | 2.79 | 2.30 | 6.36E-04 | 0.01 |
| 635 | HOXA5 | 0.05 | 21.43 | 21.38 | 7.51E-05 | 0.00 |
| 636 | RFX8 | 0.32 | 17.03 | 16.70 | 1.85E-05 | 0.00 |
| 637 | CD1C | 0.18 | 4.91 | 4.73 | 1.68E-03 | 0.02 |
| 638 | IGHD3-3 | 1.91 | 103.23 | 101.32 | 1.30E-04 | 0.01 |
| 639 | PHKA2 | 8.22 | 12.46 | 4.25 | 3.70E-03 | 0.03 |
| 640 | BEX3 | 0.57 | 14.58 | 14.00 | 1.28E-03 | 0.02 |
| 641 | FAM20C | 0.44 | 3.78 | 3.34 | 3.26E-03 | 0.03 |
| 642 | PLEKHM1P1 | 8.87 | 13.70 | 4.83 | 4.75E-03 | 0.04 |
| 643 | TLR1 | 1.81 | 8.57 | 6.76 | 7.67E-05 | 0.00 |
| 644 | ARHGAP27 | 7.41 | 14.08 | 6.68 | 2.43E-03 | 0.03 |
| 645 | ABO | 0.09 | 8.46 | 8.37 | 2.98E-04 | 0.01 |
| 646 | NACC2 | 1.72 | 5.64 | 3.92 | 2.51E-03 | 0.03 |
| 647 | ITGAX | 5.54 | 31.63 | 26.09 | 2.10E-04 | 0.01 |
| 648 | HOXA4 | 0.03 | 3.48 | 3.45 | 2.10E-04 | 0.01 |
| 649 | TRGJP1 | 4.72 | 32.24 | 27.53 | 6.53E-04 | 0.01 |
| 650 | SLC44A1 | 1.12 | 16.99 | 15.87 | 1.35E-05 | 0.00 |
| 651 | SEMA4C | 1.56 | 4.48 | 2.92 | 6.26E-03 | 0.05 |
| 652 | PF4 | 0.93 | 15.47 | 14.55 | 6.65E-03 | 0.05 |
| 653 | NKG7 | 16.93 | 69.24 | 52.31 | 2.46E-04 | 0.01 |
| 654 | SCHIP1 | 0.04 | 2.53 | 2.50 | 6.22E-05 | 0.00 |
| 655 | HNRNPLL | 0.44 | 4.06 | 3.62 | 1.25E-04 | 0.01 |
| 656 | CD101 | 0.38 | 3.71 | 3.33 | 1.47E-03 | 0.02 |
| 657 | CHST15 | 0.73 | 7.12 | 6.39 | 1.57E-03 | 0.02 |
| 658 | AC005632.2 | 2.76 | 6.83 | 4.07 | 4.90E-03 | 0.04 |
| 659 | AC066613.1 | 0.69 | 4.75 | 4.05 | 2.76E-04 | 0.01 |
| 660 | USP40 | 1.96 | 5.71 | 3.75 | 4.83E-05 | 0.00 |
| 661 | SGMS1 | 2.24 | 4.25 | 2.02 | 1.99E-03 | 0.02 |
| 662 | PLCB2 | 29.30 | 59.72 | 30.42 | 9.02E-04 | 0.01 |
| 663 | MVP | 7.62 | 15.67 | 8.05 | 3.58E-03 | 0.03 |
| 664 | AC008894.2 | 1.21 | 4.72 | 3.51 | 9.82E-05 | 0.01 |
| 665 | KCNQ1 | 0.43 | 4.95 | 4.52 | 1.30E-04 | 0.01 |
| 666 | AC010731.1 | 0.47 | 3.90 | 3.42 | 3.47E-03 | 0.03 |
| 667 | IDE | 5.71 | 8.85 | 3.13 | 1.68E-03 | 0.02 |
| 668 | NCF1B | 0.55 | 5.92 | 5.37 | 6.29E-04 | 0.01 |
| 669 | SNTB1 | 9.83 | 16.84 | 7.01 | 5.89E-03 | 0.04 |
| 670 | TOR3A | 7.67 | 10.55 | 2.88 | 4.33E-03 | 0.04 |
| 671 | APOBEC3A | 0.28 | 7.11 | 6.84 | 5.05E-04 | 0.01 |
| 672 | LILRB3 | 0.69 | 8.63 | 7.94 | 4.83E-05 | 0.00 |
| 673 | HEXB | 7.79 | 17.13 | 9.34 | 1.63E-03 | 0.02 |
| 674 | AC010768.2 | 2.93 | 8.13 | 5.21 | 1.28E-03 | 0.02 |
| 675 | PPCDC | 3.23 | 5.68 | 2.45 | 2.46E-04 | 0.01 |
| 676 | PPP1R21 | 11.48 | 14.72 | 3.24 | 4.20E-03 | 0.04 |
| 677 | MAP3K2 | 9.67 | 14.13 | 4.46 | 3.05E-03 | 0.03 |
| 678 | PRELID1 | 8.25 | 13.88 | 5.63 | 6.26E-03 | 0.05 |
| 679 | AC132872.1 | 1.90 | 4.00 | 2.10 | 1.93E-03 | 0.02 |
| 680 | HOXA10 | 0.67 | 8.64 | 7.97 | 4.60E-04 | 0.01 |
| 681 | CPNE8 | 0.10 | 8.78 | 8.68 | 2.46E-04 | 0.01 |
| 682 | CXCR2P1 | 0.16 | 3.36 | 3.19 | 1.24E-03 | 0.02 |
| 683 | CD84 | 7.85 | 28.57 | 20.72 | 4.07E-05 | 0.00 |
| 684 | DEFB1 | 0.02 | 16.65 | 16.63 | 4.57E-04 | 0.01 |
| 685 | SLC48A1 | 2.94 | 7.15 | 4.21 | 1.59E-04 | 0.01 |
| 686 | SAMD8 | 3.62 | 6.09 | 2.47 | 4.86E-04 | 0.01 |
| 687 | HOXB-AS3 | 0.01 | 5.28 | 5.27 | 4.99E-03 | 0.04 |
| 688 | GPBAR1 | 0.59 | 3.87 | 3.28 | 1.86E-03 | 0.02 |
| 689 | BLOC1S2 | 6.21 | 8.66 | 2.45 | 5.06E-03 | 0.04 |
| 690 | GFI1B | 0.48 | 13.85 | 13.36 | 4.44E-05 | 0.00 |
| 691 | ITGB7 | 2.85 | 9.74 | 6.89 | 1.99E-03 | 0.02 |
| 692 | CASP1 | 7.77 | 20.37 | 12.60 | 1.63E-03 | 0.02 |
| 693 | KCTD12 | 1.98 | 25.69 | 23.70 | 7.54E-04 | 0.01 |
| 694 | PAXX | 7.19 | 14.47 | 7.27 | 2.13E-03 | 0.02 |
| 695 | TALDO1 | 49.37 | 77.78 | 28.41 | 4.52E-04 | 0.01 |
| 696 | LINC00173 | 0.45 | 5.00 | 4.54 | 1.59E-04 | 0.01 |
| 697 | TRGC2 | 2.41 | 15.69 | 13.29 | 5.05E-04 | 0.01 |
| 698 | CTSB | 15.76 | 51.68 | 35.92 | 1.80E-04 | 0.01 |
| 699 | RAF1 | 19.43 | 24.27 | 4.84 | 1.04E-03 | 0.02 |
| 700 | PSTPIP2 | 9.90 | 20.99 | 11.10 | 5.85E-04 | 0.01 |
| 701 | TNFSF13 | 2.83 | 7.41 | 4.58 | 1.33E-03 | 0.02 |
| 702 | HOXB3 | 0.11 | 15.24 | 15.12 | 2.36E-04 | 0.01 |
| 703 | FKBP15 | 5.92 | 11.31 | 5.40 | 1.47E-03 | 0.02 |
| 704 | MADD | 19.83 | 26.70 | 6.87 | 7.05E-03 | 0.05 |
| 705 | HOXA-AS3 | 0.00 | 2.44 | 2.43 | 2.45E-04 | 0.01 |
| 706 | CPPED1 | 6.93 | 14.48 | 7.54 | 7.05E-03 | 0.05 |
| 707 | NKX2-3 | 0.00 | 4.14 | 4.14 | 1.19E-03 | 0.02 |
| 708 | BCL3 | 5.81 | 15.11 | 9.30 | 4.47E-03 | 0.04 |
| 709 | ABCA2 | 4.89 | 17.60 | 12.72 | 5.43E-04 | 0.01 |
| 710 | SULF2 | 0.90 | 10.29 | 9.38 | 4.44E-05 | 0.00 |
| 711 | RPS6KA1 | 8.95 | 29.83 | 20.88 | 1.23E-05 | 0.00 |
| 712 | ADGRE1 | 0.75 | 13.26 | 12.52 | 4.25E-05 | 0.00 |
| 713 | CHMP4B | 24.94 | 38.90 | 13.96 | 3.74E-04 | 0.01 |
| 714 | LRRC25 | 2.95 | 20.18 | 17.23 | 6.07E-03 | 0.05 |
| 715 | AC008115.3 | 2.40 | 5.73 | 3.33 | 1.52E-03 | 0.02 |
| 716 | AC062037.2 | 1.07 | 5.85 | 4.78 | 2.06E-04 | 0.01 |
| 717 | SPNS3 | 1.38 | 34.22 | 32.84 | 2.42E-05 | 0.00 |
| 718 | ZBTB18 | 5.71 | 10.84 | 5.14 | 3.47E-04 | 0.01 |
| 719 | SIGLEC17P | 0.57 | 5.66 | 5.08 | 1.66E-04 | 0.01 |
| 720 | HOXA-AS2 | 0.02 | 3.64 | 3.62 | 1.11E-04 | 0.01 |
| 721 | RAB28 | 5.03 | 7.34 | 2.31 | 2.60E-03 | 0.03 |
| 722 | RN7SKP78 | 3.76 | 7.47 | 3.71 | 5.71E-03 | 0.04 |
| 723 | LILRB2 | 0.74 | 19.77 | 19.03 | 4.83E-05 | 0.00 |
| 724 | FNIP2 | 1.42 | 3.73 | 2.31 | 3.47E-04 | 0.01 |
| 725 | IGHD1-14 | 0.51 | 44.23 | 43.71 | 1.23E-04 | 0.01 |
| 726 | ATG4D | 4.82 | 9.71 | 4.89 | 3.47E-04 | 0.01 |
| 727 | RBM47 | 0.68 | 8.48 | 7.79 | 1.28E-03 | 0.02 |
| 728 | HOXA3 | 0.02 | 6.42 | 6.40 | 5.97E-05 | 0.00 |
| 729 | CTBS | 5.09 | 8.63 | 3.54 | 3.36E-03 | 0.03 |
| 730 | LILRA6 | 0.22 | 5.04 | 4.81 | 1.41E-04 | 0.01 |
| 731 | PIP4P2 | 4.17 | 8.22 | 4.05 | 1.86E-03 | 0.02 |
| 732 | ARHGAP30 | 32.01 | 48.33 | 16.32 | 6.65E-03 | 0.05 |
| 733 | ADAM9 | 1.29 | 5.34 | 4.05 | 5.05E-04 | 0.01 |
| 734 | MFSD2A | 0.66 | 2.77 | 2.11 | 1.57E-03 | 0.02 |
| 735 | SH3BP2 | 3.74 | 18.15 | 14.40 | 8.96E-06 | 0.00 |
| 736 | IGHD5-12 | 0.70 | 91.36 | 90.66 | 3.73E-05 | 0.00 |
| 737 | CHST11 | 20.10 | 27.34 | 7.24 | 4.20E-03 | 0.04 |
| 738 | CD36 | 4.01 | 26.31 | 22.30 | 2.51E-03 | 0.03 |
| 739 | AL158207.2 | 7.60 | 13.89 | 6.28 | 2.35E-03 | 0.03 |
| 740 | AC021054.1 | 1.66 | 3.92 | 2.27 | 5.49E-05 | 0.00 |
| 741 | PKIG | 1.87 | 5.90 | 4.03 | 1.94E-04 | 0.01 |
| 742 | AC087286.2 | 1.62 | 3.82 | 2.20 | 2.77E-03 | 0.03 |
| 743 | TPCN1 | 2.97 | 9.61 | 6.64 | 1.25E-04 | 0.01 |
| 744 | AC008753.2 | 0.20 | 3.72 | 3.52 | 4.67E-04 | 0.01 |
| 745 | CRLF2 | 0.24 | 4.38 | 4.15 | 5.24E-04 | 0.01 |
| 746 | GLCE | 1.34 | 3.97 | 2.62 | 1.47E-04 | 0.01 |
| 747 | NOXA1 | 0.85 | 5.15 | 4.30 | 4.04E-04 | 0.01 |
| 748 | MYO1F | 26.91 | 51.74 | 24.83 | 4.07E-03 | 0.04 |
| 749 | AC009152.1 | 1.58 | 6.16 | 4.58 | 2.87E-04 | 0.01 |
| 750 | RNA5SP498 | 3.91 | 18.03 | 14.12 | 6.45E-03 | 0.05 |
| 751 | B3GALNT1P1 | 1.99 | 4.90 | 2.91 | 4.20E-03 | 0.04 |
| 752 | HBEGF | 2.74 | 9.41 | 6.68 | 6.26E-03 | 0.05 |
| 753 | CTSH | 3.31 | 10.07 | 6.76 | 6.65E-03 | 0.05 |
| 754 | AC010880.1 | 0.29 | 13.36 | 13.07 | 1.41E-03 | 0.02 |
| 755 | ST3GAL4 | 4.79 | 12.48 | 7.69 | 3.36E-03 | 0.03 |
| 756 | RPL34P22 | 0.47 | 2.88 | 2.41 | 6.49E-05 | 0.00 |
| 757 | FBXO7 | 15.03 | 19.95 | 4.92 | 3.36E-03 | 0.03 |
| 758 | C15orf39 | 5.15 | 31.84 | 26.69 | 1.41E-05 | 0.00 |
| 759 | AC004540.2 | 0.06 | 2.23 | 2.17 | 5.60E-05 | 0.00 |
| 760 | CPNE3 | 2.96 | 13.44 | 10.47 | 1.30E-04 | 0.01 |
| 761 | PKM | 43.42 | 83.28 | 39.87 | 2.43E-03 | 0.03 |
| 762 | ARHGAP45 | 26.84 | 50.06 | 23.22 | 1.74E-03 | 0.02 |
| 763 | AC010186.2 | 1.41 | 6.77 | 5.36 | 1.03E-05 | 0.00 |
| 764 | HOXB8 | 0.01 | 2.34 | 2.33 | 3.11E-03 | 0.03 |
| 765 | ADARB1 | 1.02 | 4.65 | 3.63 | 1.77E-05 | 0.00 |
| 766 | LGALS2 | 0.25 | 11.44 | 11.19 | 1.74E-03 | 0.02 |
| 767 | ST8SIA6 | 0.11 | 9.18 | 9.07 | 1.48E-05 | 0.00 |
| 768 | AC008753.3 | 0.14 | 3.22 | 3.08 | 3.46E-04 | 0.01 |
| 769 | SNX18P7 | 0.00 | 2.17 | 2.17 | 1.32E-03 | 0.02 |
| 770 | AC020658.5 | 1.68 | 4.94 | 3.26 | 1.02E-04 | 0.01 |
| 771 | IGHD3-22 | 0.92 | 31.20 | 30.28 | 2.97E-04 | 0.01 |
| 772 | SMARCD3 | 1.16 | 3.83 | 2.67 | 1.68E-03 | 0.02 |
| 773 | RPL7AP64 | 0.09 | 2.12 | 2.03 | 5.94E-03 | 0.05 |
| 774 | CDK11B | 7.12 | 9.55 | 2.44 | 3.82E-03 | 0.03 |
| 775 | KCTD5 | 5.05 | 7.40 | 2.36 | 5.38E-03 | 0.04 |
| 776 | MAFB | 0.47 | 18.21 | 17.75 | 2.36E-04 | 0.01 |
| 777 | SPINK2 | 0.33 | 72.23 | 71.89 | 3.14E-05 | 0.00 |
| 778 | CEMIP2 | 1.47 | 4.60 | 3.14 | 1.66E-04 | 0.01 |
| 779 | DMXL2 | 3.38 | 21.92 | 18.54 | 3.34E-04 | 0.01 |
| 780 | IFFO2 | 0.81 | 3.85 | 3.03 | 1.85E-05 | 0.00 |
| 781 | AC018529.1 | 1.28 | 3.42 | 2.14 | 3.22E-04 | 0.01 |
| 782 | DCTN3 | 6.10 | 8.26 | 2.15 | 5.54E-03 | 0.04 |
| 783 | CD163 | 0.42 | 25.83 | 25.41 | 2.35E-03 | 0.03 |
| 784 | SLC9A9 | 0.38 | 2.49 | 2.11 | 3.47E-04 | 0.01 |
| 785 | PCAT18 | 0.43 | 5.42 | 4.99 | 2.35E-03 | 0.03 |
| 786 | ANXA7 | 28.88 | 36.49 | 7.61 | 6.65E-03 | 0.05 |
| 787 | AKT3 | 0.99 | 4.66 | 3.68 | 5.71E-03 | 0.04 |
| 788 | RAB11FIP1 | 7.37 | 20.62 | 13.25 | 6.07E-04 | 0.01 |
| 789 | WNK1 | 20.99 | 30.26 | 9.26 | 1.57E-03 | 0.02 |
| 790 | CEBPA | 13.31 | 46.13 | 32.83 | 6.53E-04 | 0.01 |
| 791 | TLR6 | 1.26 | 6.04 | 4.78 | 5.26E-05 | 0.00 |
| 792 | CRYBG3 | 0.21 | 2.70 | 2.49 | 1.07E-04 | 0.01 |
| 793 | STK10 | 18.10 | 29.64 | 11.54 | 2.43E-03 | 0.03 |
| 794 | MAFG | 12.44 | 27.25 | 14.81 | 2.42E-05 | 0.00 |
| 795 | ANXA5 | 3.38 | 28.72 | 25.34 | 1.80E-03 | 0.02 |
| 796 | HOXA6 | 0.03 | 11.06 | 11.03 | 3.79E-04 | 0.01 |
| 797 | ZNF335 | 7.12 | 10.55 | 3.43 | 6.45E-03 | 0.05 |
| 798 | SLC23A2 | 5.76 | 11.60 | 5.84 | 4.63E-05 | 0.00 |
| 799 | IGHD6-25 | 0.68 | 29.60 | 28.91 | 9.12E-04 | 0.02 |
| 800 | EMB | 43.06 | 91.07 | 48.01 | 3.94E-03 | 0.04 |
| 801 | TRIM38 | 9.99 | 20.26 | 10.27 | 1.47E-04 | 0.01 |
| 802 | AJ009632.2 | 0.06 | 8.11 | 8.05 | 6.57E-03 | 0.05 |
| 803 | EAF1 | 5.63 | 8.69 | 3.06 | 1.11E-03 | 0.02 |
| 804 | SYNJ2 | 0.76 | 4.02 | 3.26 | 1.20E-04 | 0.01 |
| 805 | LIN7A | 0.03 | 2.40 | 2.36 | 2.86E-03 | 0.03 |
| 806 | AP5B1 | 7.26 | 23.88 | 16.62 | 5.43E-04 | 0.01 |
| 807 | TRGJP2 | 5.49 | 50.68 | 45.18 | 5.06E-03 | 0.04 |
| 808 | TSEN34 | 3.62 | 6.05 | 2.43 | 1.20E-03 | 0.02 |
| 809 | AC099494.3 | 0.87 | 3.22 | 2.35 | 1.07E-04 | 0.01 |
| 810 | NOD2 | 0.53 | 5.41 | 4.87 | 4.52E-04 | 0.01 |
| 811 | PRR7-AS1 | 2.40 | 5.91 | 3.51 | 6.77E-05 | 0.00 |
| 812 | C19orf38 | 3.69 | 15.09 | 11.39 | 1.15E-03 | 0.02 |
| 813 | CMAHP | 7.69 | 20.65 | 12.96 | 1.94E-04 | 0.01 |
| 814 | IQSEC1 | 4.65 | 14.16 | 9.51 | 7.02E-04 | 0.01 |
| 815 | PLBD1 | 0.63 | 22.15 | 21.52 | 8.40E-04 | 0.01 |
| 816 | SECTM1 | 0.17 | 6.27 | 6.10 | 7.99E-05 | 0.00 |
| 817 | SORT1 | 1.66 | 9.87 | 8.21 | 4.07E-03 | 0.04 |
| 818 | RNASE6 | 9.26 | 54.54 | 45.28 | 7.05E-03 | 0.05 |
| 819 | SLC46A3 | 2.03 | 6.99 | 4.96 | 1.53E-04 | 0.01 |
| 820 | AC127521.1 | 2.45 | 20.76 | 18.31 | 5.72E-05 | 0.00 |
| 821 | VSIR | 8.82 | 79.20 | 70.38 | 1.41E-05 | 0.00 |
| 822 | LAT2 | 2.44 | 46.14 | 43.70 | 8.96E-06 | 0.00 |
| 823 | ATP6V0A1 | 1.30 | 8.64 | 7.34 | 3.14E-05 | 0.00 |
| 824 | BLVRB | 4.64 | 27.22 | 22.58 | 5.97E-05 | 0.00 |
| 825 | KLF4 | 4.10 | 24.94 | 20.83 | 4.75E-03 | 0.04 |
| 826 | RNU7-61P | 1.20 | 5.57 | 4.37 | 2.60E-03 | 0.03 |
| 827 | IRF2BPL | 3.57 | 7.35 | 3.78 | 9.35E-04 | 0.02 |
| 828 | LCP1 | 92.25 | 303.44 | 211.19 | 4.25E-05 | 0.00 |
| 829 | MEFV | 0.41 | 6.89 | 6.48 | 4.75E-03 | 0.04 |
| 830 | IGHD6-6 | 0.79 | 92.63 | 91.84 | 1.40E-04 | 0.01 |
| 831 | RELT | 6.37 | 15.45 | 9.09 | 2.19E-04 | 0.01 |
| 832 | SNRPGP4 | 0.98 | 4.10 | 3.12 | 5.54E-03 | 0.04 |
| 833 | IRAK1 | 19.23 | 29.22 | 9.99 | 6.07E-03 | 0.05 |
| 834 | TICAM1 | 5.56 | 10.51 | 4.94 | 1.57E-03 | 0.02 |
| 835 | DNAJB12 | 7.56 | 9.80 | 2.24 | 4.90E-03 | 0.04 |
| 836 | CAPG | 1.26 | 37.01 | 35.75 | 8.96E-06 | 0.00 |
| 837 | ALAD | 9.68 | 15.85 | 6.17 | 6.07E-04 | 0.01 |
| 838 | CAPRIN2 | 1.02 | 4.52 | 3.50 | 6.49E-05 | 0.00 |
| 839 | TSPYL2 | 2.36 | 5.56 | 3.20 | 2.96E-03 | 0.03 |
| 840 | GCLM | 0.78 | 4.18 | 3.39 | 1.85E-05 | 0.00 |
| 841 | SNAI1 | 0.51 | 2.96 | 2.46 | 1.04E-03 | 0.02 |
| 842 | SLC8B1 | 4.84 | 9.76 | 4.92 | 9.43E-05 | 0.00 |
| 843 | CD300A | 3.89 | 19.45 | 15.56 | 5.72E-05 | 0.00 |
| 844 | AC084871.1 | 1.55 | 9.58 | 8.03 | 5.54E-03 | 0.04 |
| 845 | IL17RA | 7.71 | 36.70 | 28.99 | 1.24E-03 | 0.02 |
| 846 | IL1RN | 0.17 | 5.99 | 5.83 | 8.68E-05 | 0.00 |
| 847 | PAQR8 | 2.97 | 6.36 | 3.39 | 5.89E-03 | 0.04 |
| 848 | RN7SL328P | 2.37 | 5.76 | 3.39 | 7.02E-04 | 0.01 |
| 849 | MSRB1 | 6.62 | 15.77 | 9.15 | 4.33E-03 | 0.04 |
| 850 | LITAF | 12.90 | 29.79 | 16.90 | 1.68E-03 | 0.02 |
| 851 | SMIM25 | 0.46 | 8.35 | 7.89 | 1.93E-03 | 0.02 |
| 852 | FGL2 | 3.38 | 40.11 | 36.73 | 7.28E-04 | 0.01 |
| 853 | AC098869.1 | 0.38 | 3.25 | 2.87 | 5.38E-03 | 0.04 |
| 854 | IL6R | 11.91 | 30.09 | 18.18 | 3.60E-04 | 0.01 |
| 855 | GSR | 10.97 | 22.07 | 11.11 | 1.36E-04 | 0.01 |
| 856 | DRAP1 | 13.61 | 20.94 | 7.33 | 4.07E-03 | 0.04 |
| 857 | FAM13A-AS1 | 3.14 | 5.53 | 2.40 | 5.43E-04 | 0.01 |
| 858 | DPEP3 | 0.22 | 2.64 | 2.41 | 5.24E-04 | 0.01 |
| 859 | SIDT2 | 4.65 | 10.81 | 6.16 | 3.74E-04 | 0.01 |
| 860 | CAVIN2 | 0.38 | 8.38 | 8.00 | 9.43E-05 | 0.00 |
| 861 | CTSS | 29.40 | 118.26 | 88.86 | 1.28E-03 | 0.02 |
| 862 | QPCT | 0.27 | 3.16 | 2.89 | 4.33E-03 | 0.04 |
| 863 | ABR | 3.63 | 13.49 | 9.87 | 7.06E-05 | 0.00 |
| 864 | GBGT1 | 3.78 | 9.34 | 5.56 | 3.89E-04 | 0.01 |
| 865 | CYBB | 3.26 | 90.12 | 86.86 | 1.02E-04 | 0.01 |
| 866 | PTPN9 | 5.47 | 7.59 | 2.12 | 2.96E-03 | 0.03 |
| 867 | TET2-AS1 | 2.52 | 7.19 | 4.67 | 5.71E-03 | 0.04 |
| 868 | PLA2G4A | 3.98 | 10.04 | 6.06 | 1.00E-03 | 0.02 |
| 869 | DPEP2 | 6.49 | 17.45 | 10.96 | 1.87E-04 | 0.01 |
| 870 | SNAI3 | 3.02 | 5.54 | 2.52 | 4.20E-03 | 0.04 |
| 871 | RUNX3 | 5.90 | 20.68 | 14.78 | 1.99E-03 | 0.02 |
| 872 | FBXO33 | 6.62 | 8.72 | 2.10 | 6.45E-03 | 0.05 |
| 873 | CES1 | 0.05 | 9.07 | 9.02 | 5.62E-03 | 0.04 |
| 874 | LCP2 | 18.75 | 31.91 | 13.16 | 1.07E-04 | 0.01 |
| 875 | SLC12A4 | 3.56 | 7.26 | 3.71 | 1.94E-04 | 0.01 |
| 876 | ZC3H12A | 3.57 | 8.31 | 4.74 | 6.77E-04 | 0.01 |
| 877 | LSP1 | 2.83 | 38.93 | 36.10 | 3.43E-05 | 0.00 |
| 878 | TCF4 | 1.53 | 6.16 | 4.62 | 3.15E-03 | 0.03 |
| 879 | LINC02384 | 0.36 | 2.59 | 2.24 | 2.98E-04 | 0.01 |
| 880 | AC103702.1 | 0.01 | 2.66 | 2.66 | 2.96E-04 | 0.01 |
| 881 | GSE1 | 16.97 | 29.52 | 12.55 | 4.33E-03 | 0.04 |
| 882 | FCGR2C | 0.20 | 6.71 | 6.51 | 1.66E-04 | 0.01 |
| 883 | NLRC3 | 4.61 | 11.20 | 6.58 | 1.99E-03 | 0.02 |
| 884 | C9orf139 | 1.69 | 7.59 | 5.90 | 6.53E-04 | 0.01 |
| 885 | PIM2 | 15.65 | 30.18 | 14.53 | 6.85E-03 | 0.05 |
| 886 | S100A11 | 37.12 | 141.37 | 104.25 | 2.06E-03 | 0.02 |
| 887 | UNC93B1 | 8.39 | 21.64 | 13.26 | 3.82E-03 | 0.03 |
| 888 | RNA5SP39 | 3.41 | 8.46 | 5.05 | 4.75E-03 | 0.04 |
| 889 | LILRA5 | 0.37 | 12.81 | 12.43 | 2.46E-04 | 0.01 |
| 890 | SMAD3 | 5.21 | 10.13 | 4.92 | 2.43E-03 | 0.03 |
| 891 | HIP1 | 3.23 | 14.95 | 11.72 | 1.30E-04 | 0.01 |
| 892 | PRR7 | 1.34 | 5.29 | 3.95 | 1.30E-04 | 0.01 |
| 893 | NPC2 | 15.55 | 28.87 | 13.32 | 6.65E-03 | 0.05 |
| 894 | ANKRD44 | 5.29 | 11.93 | 6.64 | 1.73E-04 | 0.01 |
| 895 | CD44-AS1 | 3.87 | 9.81 | 5.94 | 3.36E-03 | 0.03 |
| 896 | TSPAN32 | 1.71 | 36.81 | 35.10 | 8.96E-06 | 0.00 |
| 897 | AL022328.2 | 5.23 | 7.94 | 2.71 | 6.85E-03 | 0.05 |
| 898 | LILRB4 | 0.61 | 6.94 | 6.34 | 6.26E-03 | 0.05 |
| 899 | CYP7B1 | 0.14 | 3.09 | 2.95 | 9.02E-04 | 0.01 |
| 900 | SDCBP | 24.65 | 42.60 | 17.95 | 4.20E-03 | 0.04 |
| 901 | FTH1 | 51.29 | 114.90 | 63.61 | 2.96E-03 | 0.03 |
| 902 | APOBR | 8.74 | 23.25 | 14.51 | 2.51E-03 | 0.03 |
| 903 | ULK1 | 6.77 | 13.91 | 7.14 | 3.34E-04 | 0.01 |
| 904 | RINL | 1.73 | 4.14 | 2.42 | 3.10E-04 | 0.01 |
| 905 | LY96 | 1.79 | 9.76 | 7.97 | 1.74E-03 | 0.02 |
| 906 | ERN1 | 2.68 | 5.25 | 2.57 | 1.04E-03 | 0.02 |
| 907 | AL590666.1 | 1.29 | 3.46 | 2.17 | 1.52E-03 | 0.02 |
| 908 | GSAP | 1.95 | 6.19 | 4.24 | 3.47E-04 | 0.01 |
| 909 | MBP | 3.73 | 7.33 | 3.61 | 1.02E-04 | 0.01 |
| 910 | NCF1 | 0.65 | 9.25 | 8.59 | 3.22E-04 | 0.01 |
| 911 | CYP27A1 | 0.12 | 3.50 | 3.39 | 1.20E-03 | 0.02 |
| 912 | CERK | 13.66 | 23.65 | 9.98 | 2.87E-04 | 0.01 |
| 913 | JAML | 1.22 | 14.03 | 12.81 | 3.60E-04 | 0.01 |
| 914 | AC138649.1 | 0.02 | 5.38 | 5.36 | 8.11E-05 | 0.00 |
| 915 | AL133330.1 | 3.92 | 9.18 | 5.26 | 3.82E-03 | 0.03 |
| 916 | STK17B | 19.31 | 38.93 | 19.62 | 4.33E-03 | 0.04 |
| 917 | KIF1B | 1.71 | 4.91 | 3.21 | 2.06E-03 | 0.02 |
| 918 | PPARD | 1.25 | 4.40 | 3.14 | 4.69E-04 | 0.01 |
| 919 | ADCY7 | 1.73 | 16.40 | 14.68 | 1.03E-05 | 0.00 |
| 920 | IL12A-AS1 | 0.02 | 3.62 | 3.60 | 3.74E-05 | 0.00 |
| 921 | ITGA2B | 1.27 | 10.78 | 9.51 | 5.43E-04 | 0.01 |
| 922 | ZFP36L1 | 3.87 | 18.02 | 14.15 | 2.28E-03 | 0.03 |
| 923 | NRG4 | 0.52 | 3.50 | 2.98 | 4.69E-04 | 0.01 |
| 924 | DUSP10 | 6.94 | 19.88 | 12.94 | 5.49E-05 | 0.00 |
| 925 | UNC119 | 3.30 | 6.46 | 3.16 | 4.07E-03 | 0.04 |
| 926 | CD82 | 9.02 | 28.71 | 19.68 | 1.47E-04 | 0.01 |
| 927 | JAG1 | 0.26 | 4.56 | 4.30 | 3.28E-05 | 0.00 |
| 928 | RHOH | 2.26 | 6.22 | 3.96 | 4.47E-03 | 0.04 |
| 929 | KIF21B | 3.46 | 7.10 | 3.64 | 2.51E-03 | 0.03 |
| 930 | CEBPB | 7.47 | 24.26 | 16.78 | 3.36E-03 | 0.03 |
| 931 | TTC13 | 4.33 | 6.83 | 2.50 | 3.47E-03 | 0.03 |
| 932 | AC067852.3 | 0.41 | 2.53 | 2.12 | 4.44E-05 | 0.00 |
| 933 | SLC16A3 | 1.79 | 9.14 | 7.36 | 1.41E-05 | 0.00 |
| 934 | BCL6 | 2.13 | 13.83 | 11.70 | 5.05E-04 | 0.01 |
| 935 | CDK9 | 16.99 | 31.57 | 14.58 | 1.41E-04 | 0.01 |
| 936 | MPP7 | 0.32 | 3.99 | 3.67 | 2.42E-05 | 0.00 |
| 937 | SH2D3C | 2.28 | 19.24 | 16.95 | 1.23E-05 | 0.00 |
| 938 | CCND3 | 14.99 | 35.27 | 20.28 | 2.55E-04 | 0.01 |
| 939 | AC060234.3 | 0.55 | 2.64 | 2.08 | 1.94E-04 | 0.01 |
| 940 | KIAA0513 | 0.51 | 5.47 | 4.96 | 3.14E-05 | 0.00 |
| 941 | SDHAP3 | 1.41 | 3.90 | 2.49 | 4.75E-03 | 0.04 |
| 942 | PCCA-AS1 | 0.76 | 4.18 | 3.42 | 4.86E-04 | 0.01 |
| 943 | AL138756.1 | 3.08 | 6.74 | 3.66 | 2.36E-04 | 0.01 |
| 944 | EFHD2 | 29.40 | 72.06 | 42.66 | 4.35E-04 | 0.01 |
| 945 | OLIG1 | 0.20 | 5.16 | 4.96 | 8.68E-05 | 0.00 |
| 946 | AC006960.2 | 0.28 | 3.23 | 2.95 | 3.58E-05 | 0.00 |
| 947 | TUBA4A | 2.03 | 9.88 | 7.85 | 7.54E-04 | 0.01 |
| 948 | AL024508.1 | 2.79 | 5.62 | 2.83 | 3.36E-03 | 0.03 |
| 949 | LFNG | 1.49 | 10.02 | 8.53 | 4.83E-05 | 0.00 |
| 950 | ABHD2 | 13.93 | 27.72 | 13.79 | 1.53E-04 | 0.01 |
| 951 | TAGLN | 2.50 | 5.13 | 2.63 | 4.20E-03 | 0.04 |
| 952 | CD47 | 24.15 | 39.78 | 15.62 | 3.47E-03 | 0.03 |
| 953 | RN7SL288P | 1.29 | 3.69 | 2.41 | 2.35E-03 | 0.03 |
| 954 | IGHD3-9 | 1.47 | 65.03 | 63.55 | 5.04E-05 | 0.00 |
| 955 | SELPLG | 12.83 | 80.67 | 67.83 | 1.18E-05 | 0.00 |
| 956 | IFNAR2 | 8.39 | 13.15 | 4.76 | 1.86E-03 | 0.02 |
| 957 | AC022239.1 | 0.07 | 2.49 | 2.42 | 4.33E-04 | 0.01 |
| 958 | ACSL1 | 6.82 | 24.72 | 17.91 | 2.51E-03 | 0.03 |
| 959 | AL035446.1 | 0.22 | 4.16 | 3.94 | 2.94E-03 | 0.03 |
| 960 | CCDC88B | 4.96 | 17.42 | 12.46 | 6.49E-05 | 0.00 |
| 961 | CRYL1 | 3.75 | 6.54 | 2.80 | 1.15E-03 | 0.02 |
| 962 | FAM8A1 | 5.55 | 10.62 | 5.07 | 1.25E-04 | 0.01 |
| 963 | PTPN22 | 3.61 | 16.55 | 12.93 | 1.23E-05 | 0.00 |
| 964 | MIR4802 | 0.77 | 8.06 | 7.29 | 4.88E-03 | 0.04 |
| 965 | AAK1 | 4.63 | 9.00 | 4.37 | 4.04E-04 | 0.01 |
| 966 | ELOVL6 | 0.83 | 2.95 | 2.11 | 3.26E-03 | 0.03 |
| 967 | AC011767.1 | 0.01 | 2.78 | 2.77 | 5.88E-04 | 0.01 |
| 968 | PLD3 | 14.24 | 36.49 | 22.25 | 5.89E-03 | 0.04 |
| 969 | BASP1 | 2.21 | 17.94 | 15.73 | 2.28E-03 | 0.03 |
| 970 | ACVR1 | 2.16 | 5.29 | 3.13 | 1.30E-04 | 0.01 |
| 971 | TMEM91 | 5.07 | 7.60 | 2.53 | 6.07E-03 | 0.05 |
| 972 | HDAC5 | 8.85 | 14.48 | 5.63 | 1.33E-03 | 0.02 |
| 973 | NPL | 0.80 | 4.75 | 3.96 | 2.96E-03 | 0.03 |
| 974 | PISD | 11.41 | 16.85 | 5.43 | 1.37E-03 | 0.02 |
| 975 | LINC00265 | 2.32 | 4.86 | 2.54 | 3.22E-04 | 0.01 |
| 976 | CCR1 | 1.73 | 15.98 | 14.25 | 1.15E-03 | 0.02 |
| 977 | CORO1C | 14.15 | 22.23 | 8.09 | 5.06E-03 | 0.04 |
| 978 | OTUD1 | 3.12 | 8.20 | 5.07 | 1.04E-03 | 0.02 |
| 979 | LST1 | 3.05 | 35.71 | 32.65 | 2.64E-05 | 0.00 |
| 980 | SGK1 | 0.63 | 4.21 | 3.58 | 4.61E-03 | 0.04 |
| 981 | SPECC1 | 3.74 | 7.47 | 3.73 | 2.10E-04 | 0.01 |
| 982 | RASD1 | 0.36 | 4.64 | 4.29 | 1.33E-03 | 0.02 |
| 983 | DEPP1 | 0.89 | 4.33 | 3.44 | 2.46E-04 | 0.01 |
| 984 | ARHGAP26 | 3.45 | 7.89 | 4.44 | 2.60E-03 | 0.03 |
| 985 | FOXRED2 | 3.26 | 6.55 | 3.29 | 1.74E-03 | 0.02 |
| 986 | AF064858.1 | 0.30 | 4.02 | 3.72 | 9.82E-05 | 0.01 |
